# The proteomic landscape and temporal dynamics of mammalian gastruloid development

**DOI:** 10.1101/2024.09.05.609098

**Published:** 2024-09-07

**Authors:** Riddhiman K. Garge, Valerie Lynch, Rose Fields, Silvia Casadei, Sabrina Best, Jeremy Stone, Matthew Snyder, Chris D. McGann, Jay Shendure, Lea M. Starita, Nobuhiko Hamazaki, Devin K. Schweppe

**Affiliations:** 1 Department of Genome Sciences, University of Washington, Seattle, Washington, USA; 2 Brotman Baty Institute for Precision Medicine, Seattle, Washington, USA; 3 Institute of Stem Cell and Regenerative Medicine, University of Washington, Seattle, Washington, USA; 4 Howard Hughes Medical Institute, University of Washington, Seattle, Washington, USA; 5 Seattle Hub for Synthetic Biology, Seattle, Washington, USA

## Abstract

Gastrulation is the highly coordinated process by which the early embryo breaks symmetry, establishes germ layers and a body plan, and sets the stage for organogenesis. As early mammalian development is challenging to study *in vivo,* stem cell-derived models have emerged as powerful surrogates, *e.g.* human and mouse gastruloids. However, although single cell RNA-seq (scRNA-seq) and high-resolution imaging have been extensively applied to characterize such *in vitro* embryo models, a paucity of measurements of protein dynamics and regulation leaves a major gap in our understanding. Here, we sought to address this by applying quantitative proteomics to human and mouse gastruloids at four key stages of their differentiation (naïve ESCs, primed ESCs, early gastruloids, late gastruloids). To the resulting data, we perform network analysis to map the dynamics of expression of macromolecular protein complexes and biochemical pathways, including identifying cooperative proteins that associate with them. With matched RNA-seq and phosphosite data from these same stages, we investigate pathway-, stage- and species-specific aspects of translational and post-translational regulation, *e.g.* finding peri-gastrulation stages of human and mice to be discordant with respect to the mitochondrial transcriptome vs. proteome, and nominating novel kinase-substrate relationships based on phosphosite dynamics. Finally, we leverage correlated dynamics to identify conserved protein networks centered around congenital disease genes. Altogether, our data (https://gastruloid.brotmanbaty.org/) and analyses showcase the potential of intersecting *in vitro* embryo models and proteomics to advance our understanding of early mammalian development in ways not possible through transcriptomics alone.

## INTRODUCTION

Gastrulation is a crucial process in metazoan development through which the implanted blastocyst transforms into a three germ layer structure, the gastrula^1^. Ethical and practical challenges prevent us from routinely obtaining or culturing gastrula-stage human embryos, such that our understanding of human gastrulation remains limited^2,3^. Although conserved aspects of mammalian gastrulation can be studied *in vivo* in the mouse, this also suffers from practical challenges (*e.g.* opacity, limited material, and the cost of genetic manipulation). Furthermore, mouse and human gastrulation are dissimilar in many respects. Most obviously, mouse gastrula are shaped like “egg cylinders”, while human gastrula, like most other mammals, are flat discs^4^. These morphological contrasts are accompanied by differences in the expression or source of key regulators (*e.g.* FGF8, BMP4) as well as in the origins and timing of appearance of various cell types (*e.g.* primordial germ cells, extraembryonic ectoderm)^4^.

*In vitro* stem cell-derived embryo models are powerful surrogates for *in vivo* embryos, and in recent years have proliferated not only in usage but also with respect to the specific aspects of embryogenesis that are modelled^5^. One particular class of embryo models, gastruloids, are generated by first aggregating hundreds of embryonic stem cells (ESCs) and then inducing WNT signaling, which triggers axial elongation and the emergence of all three germ layers over the ensuing days^6-8^. In the presence of extracellular matrix-like scaffolding material (“Matrigel”), mouse gastruloids form morphological structures resembling their *in vivo* counterparts, including an elongated neural tube and flanking somites^8,9^. Recently, we demonstrated that early retinoic acid, together with Matrigel, yields human gastruloids with these same morphological structures, as well as advanced cell types including neural crest, neural progenitors, renal progenitors, and myocytes (“RA-gastruloids”)^10^. Importantly, gastruloids can be chemically and/or genetically manipulated, visually and/or molecularly characterized, and, owing to their ease of culturing, even grown in large numbers^7^.

Various groups, including us, have subjected time-courses of gastruloids to scRNA-seq to characterize the dynamics of the transcriptome as ESCs diversify into germ layers and cell types^11,12^. However, RNA is only the messenger. It is proteins that are the workhorses of the cell, and in the context of differentiating gastruloids, proteins that form the structures that make emerging germ layers and cell types morphologically and functionally unique. It is challenging to accurately estimate protein abundances from transcriptomics alone^13-17^. Studies report varying levels of discordance^18-22^, *e.g.* one recent study found that in human cells, transcript abundance accounted for only ~40% of the variance in protein levels^23^. Moreover, post-translational modifications (PTMs) including phosphorylation, ubiquitination, and glycosylation vastly increase the functional diversity of cells’ proteomes to over ~10 million proteoforms^24^, aspects of identity and function that are entirely absent from a transcriptomic census. Such PTMs are known to dynamically regulate signaling pathways that critically underpin developmental patterning and cell type specification, *e.g.* WNT, BMP and FGF^25^. However, only a handful of studies to date have attempted to characterize the proteome in early mammalian developmental contexts, and, to our knowledge, none in human post-implantation embryos or gastruloids^13,26^.

Here we applied high-throughput quantitative mass spectrometry to quantify proteins and phosphosites across four key stages of gastruloid differentiation. With these data, we map the dynamics of hundreds of known protein complexes, while also identifying additional proteins whose temporal profiles correlate with specific complexes, suggesting cooperative relationships during early development. With experimentally matched RNA-seq data, we identify pathway-specific patterns of concordance and discordance between the transcriptome and proteome during gastrulation. Extending our study to phosphorylated proteins, we also map the dynamics of thousands of phosphosites and predict stage-specific kinase activities across gastruloid development. Finally, by profiling the dynamics of proteins associated with developmental disorders, we identify their cooperative partners, interaction networks and phosphosite dynamics. Altogether, by focusing on the proteome and phosphoproteome in models of early gastrulation, these data and analyses lay the groundwork for closing the gap between transcriptomic vs. cellular views of early mammalian development. The data are made freely available together with a custom browser at: https://gastruloid.brotmanbaty.org/.

## RESULTS

### Quantifying the dynamic proteome from ESCs to gastruloids

**Figure 1.**
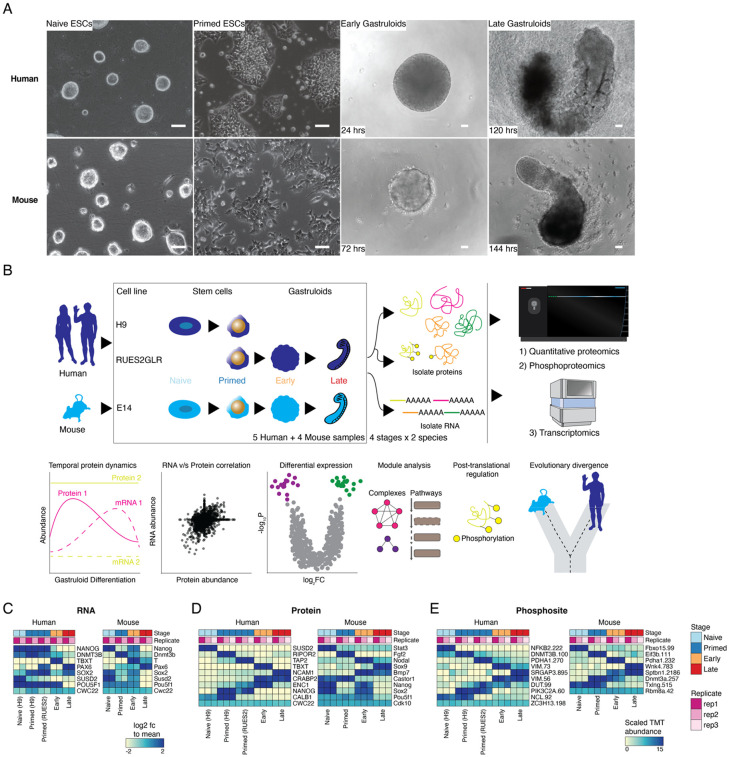
Quantifying the dynamic proteome from ESCs to gastruloids. **(A)** Representative brightfield images of human RA-gastruloids and mouse gastruloids imaged over the course of their development. Scale bar: 10 μm. **(B)** Multi-omics profiling workflow. We sampled two human cell lines (H9 and RUES2-GLR) and one mouse cell line (E14) at the indicated stages. **(C-E)** Representative heatmaps depicting the temporal dynamics of RNAs **(C),** proteins **(D),** or phosphosites **(E)** for selected developmental marker transcripts, proteins or PTMs, respectively, across replicates and stages for both human and mouse. Color scale for RNAs indicates log2-fold change relative to the row mean. Color scale for protein and phosphorylation data indicates scaled TMT abundance.

We sought to profile the dynamics of RNA, protein, and phosphosite levels in human RA-gastruloids^10^ and conventional mouse gastruloids^9^. Specifically, we performed matched bulk RNA-seq, quantitative proteomics and quantitative phosphoproteomics on whole cell extracts from both human and mouse samples corresponding to four stages of gastruloid differentiation, including two ESC stages (“naïve” and “primed”) and two gastruloid stages (“early” and “late”) (Fig. 1A; Sup. Fig. 1A). We focused on these four stages because they model pre-implantation, post-implantation, post-symmetry breaking, and anterior-posterior (A-P) elongation/patterning, respectively. For human primed ESCs, we analyzed two cell lines (H9, RUES2-GLR) to assess inter-cell-line variation^27,28^ (Fig. 1B). As such, we analyzed nine sample types altogether—four mouse (naïve ESCs, primed ESCs, early gastruloids, late gastruloids) and five human (naïve H9 ESCs, primed H9 ESCs, primed RUES2-GLR ESCs, early RUES2-GLR gastruloids, late RUES2-GLR gastruloids)—in biological duplicate (transcriptomics) or triplicate (proteomics, phosphoproteomics) (Sup. Fig. 1B-C).

To assess data quality, we calculated pairwise correlations between biological replicates of each sample type and confirmed high reproducibility for each data type (RNA: *r* > 0.98; protein: *r* > 0.93; phosphosite: *r* > 0.97; Sup. Fig 1D-F). Consistent with that, replicates for each data type were tightly grouped by Principal Components Analysis (PCA) (Sup. Fig. 1G). In human, for all three data types, PC1 separated naïve H9 ESCs from other samples (RNA: 43%; protein: 45%; phosphosite: 52% of variance explained) while PC2 broadly correlated with developmental progression (RNA: 26%; protein: 34%; phosphosite: 21% of variance explained). In mouse, for all three data types, PC1 separated late gastruloids from other samples (RNA: 56%; protein: 50%; phosphosite: 44% of variance explained), while PC2 once again resolved developmental progression (RNA: 26%; protein: 31%; phosphosite: 33% of variance explained) (Sup. Fig. 1G).

Across all replicates of all stages, we detected and quantified 7,352 human and 8,699 mouse proteins (Sup. Fig. 1C; Sup. Table 1). To gauge the depth of proteome sampling, we mapped these proteins onto the Human Protein Atlas^29^, and found all 34 annotated subcellular locations to be represented (Sup. Fig. 1H). To confirm that we are capturing developmental transitions, we searched for stage-specific markers at both the RNA and protein levels in each species. Results were generally consistent with expectation, *e.g.* in human samples, classic pluripotent markers *NANOG* and *POU5F1* were highly expressed in ESCs, while *TBXT,* a marker of gastrulation and/or mesendoderm differentiation^30^, and *PAX6,* associated with neural tube differentiation^31^, were upregulated in early and late stage gastruloids, respectively (Fig. 1C). Many detected proteins also exhibited stage specificity. For example, again focusing on human samples, SUSD2, a cell-surface marker for the naïve epiblast^32^, was only detected in naïve H9 cells, while TBXT and NCAM1 were specific to early and late stage gastruloids, respectively (Fig. 1D). Interestingly, CRAPBP2, a retinoic acid binding protein^33^, was detected only in human samples after addition of retinoic acid into the culture media^34^. For some markers, we observe consistent dynamics for mRNA and protein abundance, *e.g.* mouse *Sox2*/Sox2 (Fig. 1C-D). Using sample multiplexed quantitative proteomics^17,35^, we also quantitatively profiled 1,209 human and 1,603 mouse phosphosites (Fig. 1E) and mapped their temporal dynamics across the four stages. For example, phosphorylation of DNMT3B Ser100 (human) and Dnmt3a Thr257 (mouse) are decreased in naïve ES cells, suggesting that their phosphorylation may be associated with DNA hypomethylation in ground state pluripotency and increased methylase activity during differentiation^25,36-40^. Taken together, these anecdotes, together with the high reproducibility across biological replicates, suggest that the data are of high quality and can be leveraged to study temporal trends in RNA, protein and phosphosite levels in these models of human and mouse gastrulation.

### Time-resolved proteomics reveals biologically coherent shifts across gastruloid development

**Fig. 2.**
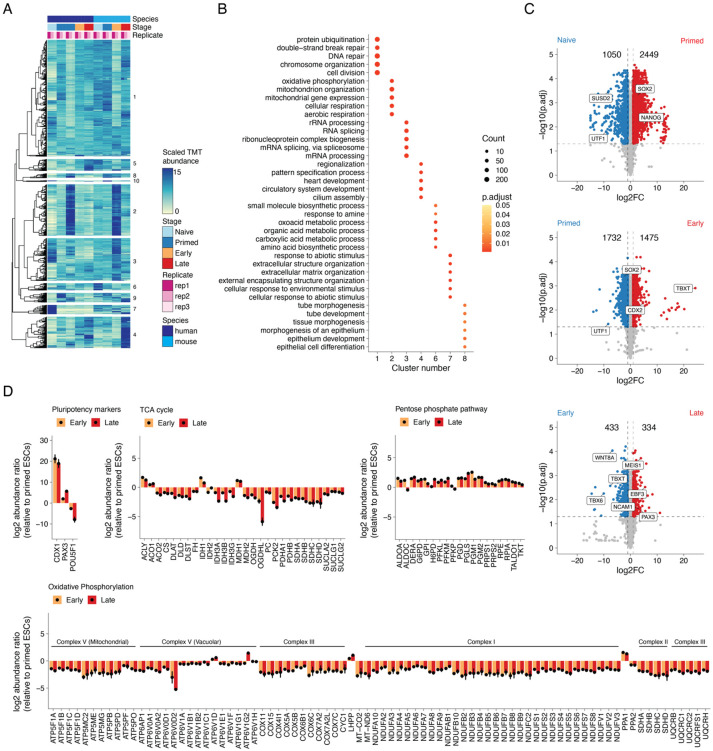
Time-resolved proteomics reveals biologically coherent shifts across gastruloid development. **(A)** Heatmap depicting the temporal dynamics of protein expression across human and mouse gastruloid differentiation samples and replicates. **(B)** Dotplot indicating the top Gene Ontology (GO) terms enrichment across clusters. Clusters 5, 9, and 10 did not have significantly enriched GO terms. Color scale indicates the Benjamini Hochberg (BH) adjusted p-values. Size of dots corresponds to the number of proteins associated with a particular GO term. **(C)** Volcano plots of the protein expression changes across consecutive stages of human gastruloid differentiation, where x-axis represents the log2 fold change between two adjacent timepoints and y-axis represents the negative log_10_ of the Benjamini-Hochberg-adjusted p-value. **(D)** The log_2_ protein abundance ratio of early (yellow) or late (red) gastruloids compared to primed human ESCs (RUES2-GLR) for proteins associated with pluripotency and central metabolism including TCA cycle, pentose phosphate pathway and oxidative phosphorylation. Mean abundance ratios are indicated with dots and error bars represent the standard deviation.

To identify sets of proteins with similar temporal dynamics, we merged the human and mouse proteomic datasets by orthology and subjected them to hierarchical clustering (Fig. 2A). Focusing on 10 clusters, *i.e.* sets of proteins that exhibit similar dynamics across both species, we assessed Gene Ontology (GO) enrichments^41^. Seven of the 10 clusters returned significantly enriched biological processes, *i.e.* cell division and DNA repair (cluster 1), mitochondria and aerobic respiration (cluster 2), RNA biogenesis (cluster 3), cilia and pattern specification (cluster 4), small molecule metabolism (cluster 6), extracellular matrix organization (cluster 7) and tube development (cluster 8) (Fig. 2B; Sup. Table 2). These enrichments suggest that the abundance of proteins that underlie these biological processes are coordinated during gastrulation.

To identify the changes in protein abundance that may underlie specific transitions in gastruloid development, we performed differential expression analysis across adjacent timepoints in each species, which identified thousands of differentially expressed proteins (DEPs) (Sup. Fig. 2A). For example, when comparing naïve and primed states of pluripotency within human H9 cells, we identified 3,499 DEPs. Among these was SUSD2, whose expression marks pre-implantation epiblasts in human blastocysts, which was detected only in the naïve state, as well as SOX2 and NANOG, which were enriched in the primed state (Fig. 2C). GO analysis of naïve vs. primed DEPs found that naïve cells were enriched for proteins involved in extracellular matrix (ECM) organization, while primed cells were enriched for proteins involved in nucleotide metabolism (Sup. Fig. 2D). In comparing primed RUES2-GLR ESCs to early human RA-gastruloids, we identified 3,207 DEPs, including SOX2 enrichment in primed ESCs, and TBXT and CDX2 enrichment in early human RA-gastruloids. Upon GO analysis, DEPs upregulated in early gastruloids mapped to actin filament organization and cytoskeletal processes, while DEPs downregulated mapped to mitochondrial processes (Sup. Fig. 2D). In comparing early vs. late human RA-gastruloids, we identified 767 DEPs, including downregulation of TBXT, caudal axial progenitors marker WNT8A, and presomitic mesoderm marker TBX6, and upregulation of markers of advanced cell types including PAX3 (dorsal somites and neural tube), SOX1 & SOX2 (neural tube) and cardiomyocytes (MEIS1) (Fig. 2C). Upon GO analysis, DEPs upregulated in late gastruloids mapped to organ morphogenetic processes and body pattern specification, while DEPs downregulated mapped to small molecule transport (Sup. Fig. 2D).

We also compared H9 vs. RUES2-GLR human primed ESCs and detected 3,047 DEPs (Sup. Fig. 2B). While both cell lines expressed detectable levels of characteristic primed ESC markers (*e.g.* SOX2, NANOG), DEPs largely mapped to mitochondrial processes (respiration, oxidative phosphorylation), which are upregulated in primed RUES2-GLR relative to primed H9 ESCs. Conversely, DEPs upregulated in primed H9 ESCs were enriched for cytoskeletal processes and translation (Sup. Fig. 2C). This comparison reinforces the view that there are substantial differences between these widely used human ESC lines^42^.

As such, the proteomes of primed RUES2-GLR ESCs were highly enriched for mitochondrial processes, relative to both primed H9 counterparts as well as RUES2-GLR-derived early RA-gastruloids (Sup. Fig 2C-D), with the latter suggesting that these processes are downregulated over the course of gastruloid differentiation. We sought to ask if this downregulation was specific to a subset of mitochondrially mediated metabolic pathways, as opposed to being more general. For this, we compared primed human ESCs vs. early and late RA-gastruloids (all RUES2-GLR-derived) with respect to individual proteins, broken down by pathway. Intriguingly, we observed highly consistent levels of downregulation of mitochondrial proteins involved in the TCA cycle and oxidative phosphorylation, and upregulation of proteins involved in the pentose phosphate pathway (Fig. 2D). Within oxidative phosphorylation, this consistency extended to individual protein complexes, *e.g.* the levels of vacuolar subunits of the ATPase complex remained relatively stable unlike their mitochondrial counterparts (Fig. 2D). These observations suggest that shifts in the levels of mitochondrial machinery are highly coordinated during gastruloid differentiation, consistent with previous studies of the remodeling of metabolic complexes across multiple organ systems during mammalian aging^17^.

Upon extending such analyses to the mouse data, we observed similar numbers of DEPs (Sup. Fig. 2E), as well as stage-specific patterns that broadly matched expectation, *e.g.* pluripotency markers Sox2 and Nanog highly expressed in naïve mESCs compared to their primed counterparts. Similarly, when comparing primed mESCs vs. early mouse gastruloids, we observed upregulation of the mesenchymal cell marker Bmp7, and in comparing early vs. late mouse gastruloids, upregulation of endoderm marker Sox17, in the more differentiated sample. To systematically analyze conserved protein expression dynamics, we compared fold-changes across pairwise stage transitions for orthologous human and mouse proteins. While we observed modest positive correlation in the naïve to primed (r_Pearson_ = 0.17) and early to late transitions (r_Pearson_ = 0.5), there was strong anticorrelation in the primed to early transition (r_Pearson_ = −0.8). However, this anticorrelation appears to be driven by the aforereferenced elevated levels of mitochondrial proteins in primed RUES2-GLR ESCs, *i.e.* the metabolic state of primed human RUES2-GLR ESCs is better matched to that of early mouse gastruloids than that of mouse primed ESCs (Sup. Fig. 2F).

### Co-regulation analysis maps cooperative protein associations to known protein complexes and pathways

The vast majority of proteins quantified here do not map to known protein complexes nor are they assigned specific biological functions during early human development. Given our observations that proteins assigned to cellular modules are coherently regulated across gastruloid development (Fig. 2D), we sought to explore such co-regulation at a more granular level, *e.g.* among members of a specific signaling pathway or protein complex. Functional proteomics has emerged as a powerful method to annotate and assign roles to proteins in understudied contexts^43,44^. Co-regulation analysis, based on calculating correlations of protein abundances in a pairwise fashion across experimental samples, can elucidate coordinated protein functions such as macromolecular complexes and biochemical pathways^45-49^. Correlated and anticorrelated edges within the resulting networks can reveal co-regulatory effects including direct protein interactions^50^, mechanisms of action for signaling networks^51,52^, and cell state-specific roles^53^.

**Figure 3.**
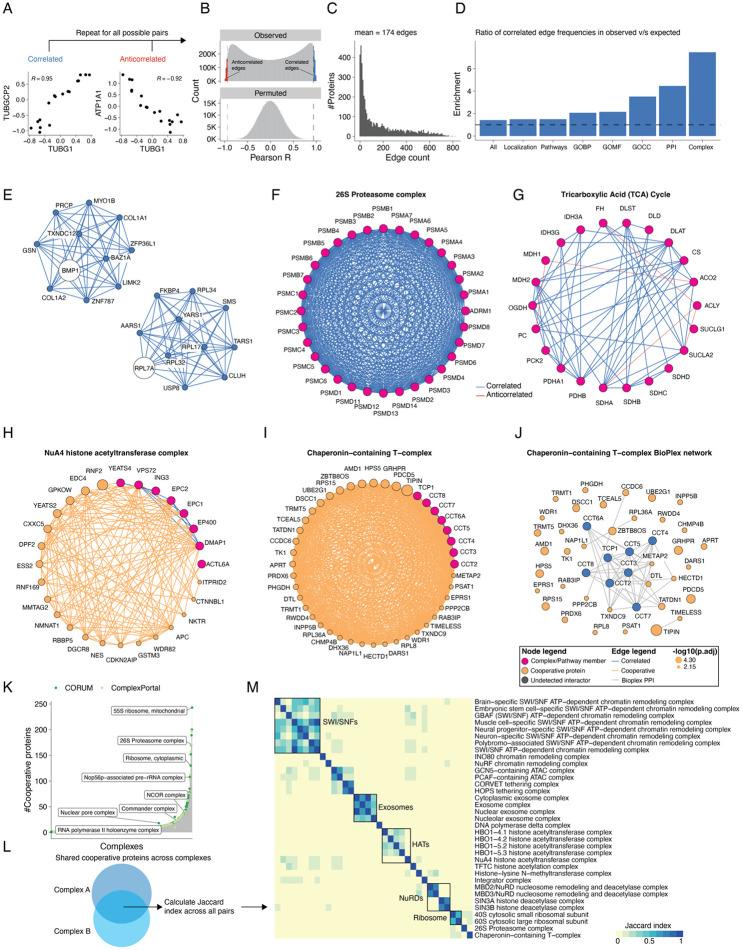
Co-regulation analysis maps cooperative protein associations to known protein complexes and pathways. (**A**) Scatterplots comparing abundances across selected protein pairs across samples. **(B)** Distribution of r_Pearson_ based on observed (top) and permuted (bottom) data. Observed distribution was obtained by calculating r_Pearson_ across all possible protein-protein pairs. Permuted distributions were generated by randomly sampling 50,000 protein pairs after randomly shuffling their respective timepoints 10 times each prior to calculating r_Pearson_. Colors indicate strongly correlated (>=0.95; blue) or anticorrelated (<=−0.95; red) edges. **(C)** Distribution of protein edge counts across the trimmed correlation network. On average, each protein in the network participated in 174 edges. **(D)** Ratio of enrichment for the annotated edges in the correlation network (“observed network”) compared to the expected edge annotation frequencies across Gene Ontology biological process (GOBP), cellular component (GOCC), molecular function (GOMF), localization, pathways, protein-protein interactions (BioPlex) or protein complexes. Specifically, we calculated the enrichment for annotated edges as the fraction of annotated edges per category in the observed correlation network divided by the fraction of annotated edges among all possible edges involving the 5,227 proteins in the correlation network. The expected frequency of annotated edges was calculated by generating all possible pairs from 5,227 human proteins (Uniprot, 07/2024) and computing the number of pairs explained by each functional category. **(E)** Network analysis identifies known associations between proteins for BMP1 and RPL7A. **(F-G)** Network structure of the **(F)** 26S proteasome and **(G)** Citric Acid cycle pathway. Magenta nodes indicate known complex members annotated either from CORUM or EMBL ComplexPortal for protein complexes, or from BioCarta, KEGG, Protein Interaction Database (PID), Reactome, and WikiPathways (WP) for biochemical pathways. Blue edges indicate positive correlations between nodes while red edges indicate anticorrelations. **(H-I)** Cooperative proteins are highly correlated with members of established protein complexes including: **(H)** NuA4 chromatin remodeling complex and **(I)** Chaperonin-containing T (TRiC/CCT) complex. Magenta nodes indicate subunits of a given complex, while orange nodes indicate cooperative proteins *i.e.* proteins with correlated profiles to proteins constituting a particular protein complex. Cooperative node sizes indicate the negative log10 of the BH-adjusted p-value after computing significance from Fisher’s exact tests to determine cooperative association of a protein to a particular module. Blue edges indicate correlated edges while orange edges link cooperative proteins to members of a particular module. **(J)** Bioplex interaction network of the TRiC/CCT complex. Orange nodes are cooperative proteins with correlated profiles to proteins found in the TRiC/CCT complex. Gray edges indicate BioPlex evidence. **(K)** Histogram of protein complexes (x-axis) and their respective numbers of cooperative proteins (y-axis). **(L)** Heuristic to identify shared cooperative proteins between complexes. **(M)** Heatmap depicting a subset of shared cooperative proteins across manually curated EMBL ComplexPortal protein complexes namely exosomes, SWI/SNFs, ATAC remodelers, nucleosome remodelers (NuRDs), and histone acetyltransferase (HAT) and deacetylase (HDAC) complexes. Heatmap colored by Jaccard similarity coefficients calculated from overlapping sets of cooperative proteins between protein complex pairs and clustered using euclidean distances with average linkage.

To apply co-regulation analysis to our data, we calculated correlations (r_Pearson_) between all 19.6 million possible pairs of the 6,261 proteins that were successfully detected and quantified in all 18 primed ESC or gastruloid samples ([3 human RUES2-GLR stages + 3 mouse stages] x 3 biological replicates). For example, proteins within known complexes were highly correlated, *e.g.* TUBG1 and TUBGCP2, which constitute the -tubulin ring complex^54^, were highly correlated, while TUBG1 was anticorrelated with the Na+/K+ transporting ATPase, ATP1A1 (Fig. 3A). Across all pairs, we observed a bimodal distribution of r_Pearson_, while a similar analysis after permuting sample IDs for individual proteins yielded a normal distribution of r_Pearson_ centered at zero (Fig. 3B).

We focused on pairs that were either strongly correlated (r_Pearson_ >=0.95) or anticorrelated (r_Pearson_ <= −0.95) at a false discovery rate (FDR) of 1% (Fig. 3C). The resulting network consisted of 5,681 nodes (proteins) and 489,417 significant correlations or edges, of which 62% were positively and 38% were negatively correlated (174±195 edges per protein; Fig. 3C; Sup. Fig. 3A-B; Sup. Table 3). We subsetted our network to 5,227 proteins by retaining only the canonical Uniprot protein isoforms detected in our datasets and validated positively correlated edges by mapping the resulting network onto the databases cataloging known gene ontologies^41^, subcellular localizations^29^, biochemical pathways^55-59^, protein-protein interactions^60^ and protein complexes^61,62^. The proportion of annotated edges that were positively correlated edges varied by database, *e.g.* 73–92% for proteins with shared GO annotations, subcellular localization or pathway databases, but 93% for proteins previously reported to interact, and 97% for proteins belonging to the same complex (Sup. Fig. 3C; Sup. Table 4).

Of the positively correlated edges in the trimmed network, 37.8% were explained by at least one established annotation, a 1.4-fold enrichment over the 26.7% of all possible edges involving these 5,227 proteins that are annotated in these databases (Sup. Fig. 3D-E). This was consistent with previous studies that attributed 34–42% of protein correlation network edges to previous annotations. Notably, those studies also required 41–375 different cell lines to generate co-regulation networks^50,53^. However, specific categories of annotation were much more enriched than others. For example, our network’s edges were only modestly enriched for shared subcellular localization (1.5-fold), but were strongly enriched for annotated protein-protein interactions (4.5-fold) and shared membership in a protein complex (7.4-fold) (Fig. 3D).

Given the relatively high proportion of positively correlated edges corresponding to protein-protein interactions and macromolecular complexes, we leveraged the untrimmed network to map positively protein pairs to specific developmental genes or protein complexes (Sup. Fig. 3E-F). Anecdotally, many known protein-protein interactions were recovered. For example, BMP1, the metalloprotease that plays roles in the formation of the extracellular matrix including the processing of procollagens to their active fibril forms^63^, was highly correlated with collagens COL1A1 and COL1A2, while RPL7A, a constituent of the large ribosomal subunit, was highly correlated with other members of the large ribosomal subunit as well as tRNA synthetases (AARS1, TARS1, YARS1) that charge tRNAs with their cognate amino acids prior to translation (Fig. 3E).

To systematically ask whether the correlation network recovered known protein complexes, we focused on 1,357 complexes from CORUM^61^ or ComplexPortal^62^ with 3+ subunits represented in our correlation network. For this subset, an average of 80% of complex members were represented among the 5,681 proteins in the network (Sup. Fig. 3G). For example, 29 of 33 (88%) of 26S proteasome complex proteins were represented, with 87% of all possible edges among these proteins detected, 100% of which were positively correlated (Fig. 3F). Similar trends were observed for core metabolic modules, including in the citric acid cycle, for which 90% of edges connecting pathway members were positively correlated, with only ACO1 and ACLY participating in anticorrelated edges (Fig. 3G).

In addition to recovering previously supported protein-protein relationships (37.8% of filtered network, Fig. 3D), we also nominated potentially novel relationships. Many of these novel edges are potentially driven by aspects of cell state that are unique to gastruloids and early development relative to the steady states of the workhorse cell lines that are the primary source material for the databases to which we compared our network^50,53^. Drawing from previous high-throughput proteomics studies^47,60^, we defined a protein cooperativity metric to enrich for first-degree neighbors of known complexes and pathways, termed “cooperative edges” (Sup. Fig. 3H). To evaluate this approach, when members of a complex were withheld from our analysis, our cooperative edge mapping framework should recover their association to the remaining protein complex network. For example, when we divided ribosomal proteins into 60S large and 40S small ribosomal subunit groups and asked which proteins were cooperatively associated with the 40S small ribosomal subunit, we find that among the top 5 most significant hits are the 60S complex members RPL5, RPL13A and RPL32 (Sup. Fig. 3H).

With this framework, we identified 1,385 cooperative proteins associating with 218 ComplexPortal complexes^62^ and 1,944 cooperative proteins associating with 524 CORUM complexes^61^ (Sup. Table 5). The number of cooperative proteins per complex varied widely and was not correlated with the number of complex subunits (Fig. 3H-K; Sup. Fig. 3I). The number of complexes that a given protein is cooperatively associated with also varied widely (Sup. Fig. 3J). When comparing cooperative protein-complex relationships with protein-protein interaction databases including BioGrid and BioPlex network of interactors^60^, we found that 1,610 cooperative edges (involving 18.5% of cooperative proteins) were annotated as direct physical interactions (Sup. Fig. 3K). An illustrative example involves the Chaperonin-containing T (TRiC/CCT) complex, for which 5 (13%) of the 36 most significantly cooperative proteins were primary BioPlex interactors, and 9 (25%) were BioGrid interactors (Fig. 3I-J). In summary, cooperative protein analysis recovered both known physical interactions as well as potentially novel associations between complexes and cooperative proteins.

We reasoned that overlaps of cooperative proteins shared by multiple complexes might inform these proteins’ functional roles. To quantify such sharing, we calculated Jaccard similarity coefficients between pairs of complexes—a perfect overlap of cooperative proteins between a given pair of complexes would result in a Jaccard similarity of 1 (Fig. 3L). Although most pairwise comparisons yielded little to no overlap in cooperative proteins, those that did were highly structured (Fig. 3M). For example, exosome complexes and histone acetyltransferase complexes each largely exhibited discrete sets of cooperative proteins that overlapped with one another but not with other complexes. The 40S and 60S ribosomal subunits, while sharing extensive overlap in terms of their cooperative proteins, also shared overlaps with the 26S proteasome and the TRiC/CCT complex (Fig. 3M; Sup. Table 6). Other subsets of protein complexes exhibited varying degrees of sharing. For example, all SWI/SNF complexes shared cooperative proteins with other SWI/SNF complexes, but a subset of these also shared cooperative proteins with tethering complexes, as well as the ATAC (Ada-two-A-containing) coactivator^64^ and histone methyltransferase complexes (Fig. 3M).

### Gastruloid stages and gene modules exhibit varying degrees of RNA-protein discordance

Previous studies spanning various biological contexts have reported varying extents of concordance between mRNA and protein levels^15,16,50,65,66^. Using the matched bulk RNA-seq data we acquired for these same samples, we next sought to assess the extent to which transcript abundances were predictive of protein levels in developing gastruloids. As noted above, our transcriptome data matched expectation with respect to temporal trends and stage-specific markers (Fig. 1D). Of note, HOX genes^67^ turned on with gastruloid induction in both species, both at the early stage in human gastruloids and the late stage in mouse gastruloids (Sup. Fig 4A).

**Figure 4.**
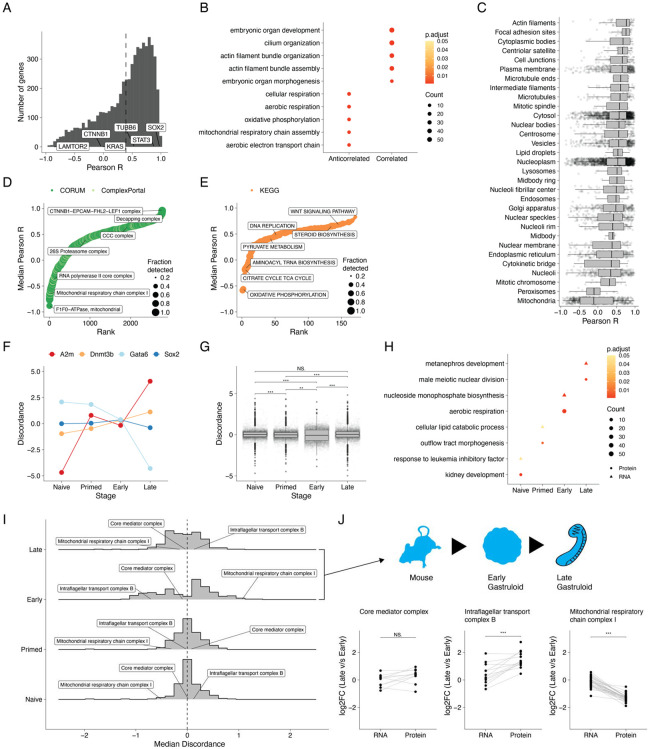
Gastruloid stages and gene modules exhibit varying degrees of RNA-protein discordance. **(A)** Histogram of correlations (r_Pearson_) between protein and RNA expression for all genes detected at the transcript and protein level in our samplings of human and mouse gastruloid development. Dashed line indicates the mean r_Pearson_ across all genes. Representative genes with varying extents of correlation are highlighted. **(B)** GO term dotplot highlighting GO-defined biological processes exhibiting high RNA-protein correlation (r_Pearson_ >= 0.75) or anticorrelation (r_Pearson_ <= −0.75). **(C)** Boxplot depicting the distribution of protein-RNA correlation (x-axis) as a function of subcellular location (y-axis). Rank plots of median RNA-protein r_Pearson_ across **(D)** protein complexes or **(E)** biochemical pathways. Colors indicate databases from which the module sets were curated. **(F)** Representative examples of RNA-protein discordance profiles (for any given gene, mean across replicate is shown) for various stages. **(G)** Boxplot depicting the distributions of RNA-protein discordances (for any given gene, mean across replicates is shown) for various mouse stages. **(H)** Dotplot highlighting the biological processes significantly enriched in genes exhibiting protein-abundant (circles; discordance >=1) or RNA-abundant (triangles; discordance <=−1) RNA-protein discordance. Color scale indicates the p-value adjusted using the Benjamini-Hochberg procedure and sizes of dots indicate the number of genes detected within each term. **(I)** Median RNA-protein discordances of members of protein complexes at each stage of mouse gastruloid development. **(J)** Comparison of the RNA and protein log_2_-scaled fold-changes between early vs. late mouse gastruloids in the Mediator complex (left), intraflagellar transport complex B (middle), and mitochondrial Complex I of the oxidative phosphorylation pathway. Significance testing on RNA and protein distributions was performed using a standard t-test.

We next calculated RNA-protein correlations of individual genes. Across the 6,010 genes with both protein and RNA data for all sampled timepoints in both species, Pearson correlation coefficients were biased towards positive correlation, consistent with previous work^50^ (mean r_Pearson_ = 0.39; Fig. 4A; Sup. Table 7). When highly correlated (r_Pearson_ >=0.75) or anticorrelated (r_Pearson_ <=−0.75), RNA-protein relationships were stratified by broad gene classes^68-71^, *e.g.* genes associated with transcription (*e.g.* SOX2) tended to be positively correlated while those associated with the ribosome (*e.g.* NSA2) tended to be anticorrelated (Sup. Fig. 4B-C). At the level of Gene Ontology (GO) biological processes, genes exhibiting highly positive RNA-protein correlation in our dataset were enriched for cytoskeletal and organ morphogenesis terms, suggesting that RNA levels are a reasonable proxy for protein abundance for these processes (Fig. 4B; Sup. Table 7). Drilling down further to the level of complex and pathways, complexes involved in transcription (*e.g.* SOX2-OCT4 complex, CTNNB1-EPCAM-FHL2-LEF1 complex and the mRNA decapping complex) and signaling pathways (WNT and MAPK signaling) tended to be positively correlated (Fig. 4D-E).

On the other hand, at the level of GO biological processes as well as shared subcellular localization (Human Protein Atlas^29^), mitochondrial genes, particularly those involved in the electron transport chain and oxidative phosphorylation, tended to have anticorrelated RNA and protein levels (Fig. 4B-C), consistent with post-transcriptional and post-translational control of mitochondrial protein levels during development^72,73^. Once again drilling down further, this trend was driven by mitochondrial protein complexes (*e.g.* F1-F0 ATPase and complex I) and pathways involved in central metabolism (*e.g.* TCA cycle and oxidative phosphorylation) (Fig. 4D-E; Sup. Table 8). In the case of Complex I, previous work in HeLa cells^74^ demonstrated that proteins in this complex were rapidly degraded post-translationally, suggesting that these systems are regulated in a similar fashion during gastruloid development.

We next sought to better understand the relationship between RNA and protein abundance as a function of developmental stage. Across all genes within each stage, we found that early mouse gastruloids exhibited substantially lower RNA-protein correlation than all other human or mouse stages (r_Pearson_ = 0.26; Sup. Fig. 4D). We defined a metric of discordance between RNA and protein measurements—the log2 transformed ratio of the average fold change of a protein to its corresponding RNA—at a given stage of gastruloid development. Thus, discordance values close to 0 signify comparable levels of RNA and protein, positive discordance implies that the protein is more abundant than its corresponding transcript and vice versa. Focusing on mouse gastruloids (where for our data, all stages arose from the same cell line), Gata6 discordance was high at the naïve ESC stage (higher than expected protein, given RNA levels); while in late gastruloids, Gata6 protein-RNA discordance was low (Fig. 4F). In contrast, SOX2 transcript and protein abundance remained relatively consistent over time (Fig. 4F).

Overall, we observed varying profiles of discordance across mouse gastruloid development (Fig. 4G, Sup. Fig. 4E). Applying GO enrichment analysis to genes with absolute discordance ratios greater than 1 (*i.e.* protein either highly more or less abundant than expected, given RNA levels), we observed that mitochondrial and metabolic processes tended to be discordant mainly in early mouse gastruloids (Fig. 4H; Sup. Table 9). To understand discordance at the gene module level, we calculated the median RNA-protein discordance among genes belonging to a particular protein complex. The distributions across the four developmental states were centered at 0 (Fig. 4I). We next compared the fold changes of RNA and proteins between two temporally adjacent stages with the aim of delineating when discordance emerges or resolves (Fig. 4J). We found that most complexes had no significant differences in discordance between stages (*e.g.* core Mediator complex, Fig. 4J). However, a minority did, *e.g.* 12% (33/279) of the protein complexes analyzed exhibited significantly different RNA and protein fold changes when comparing early vs. late stages of mouse gastruloid development. These included cytoplasmic and mitochondrial ribosomal subunits, intraflagellar transport complex B, and Complex I of the oxidative phosphorylation pathway (Fig. 4J; Sup. Fig. 4F).

Finally, we sought to assess whether the protein levels of developmental transcription factors (TFs) could be used to adjudicate potential targets (Sup. Fig. 4G). For this, we focused on Sox2, Sox3, Tfap2c, and Gata6, which exhibit distinct patterns of stage-specific protein expression during mouse gastruloid differentiation (Sup. Fig. 4H). Anecdotally, transcripts for established targets of each of these TFs were indeed upregulated in a corresponding pattern, *e.g. Nanog* with Sox2, *Top2a* with Sox3, *Dppa3* with Tfap2c, and *Sox17* with Gata6 (Sup. Fig. 4I)^75-80^. Although each of these TF has thousands of targets according to databases such as TFlink^81^, the RNA levels of only a subset of these are well-correlated with the TF’s protein levels in our data (r_Pearson_ >= 0.9), *e.g.* 582 for Sox2 (3.4% of its targets), 122 for Sox3 (3.4% of its targets), 218 for Tfap2c (3.4% of its targets), and 347 targets for Gata6 (3.4% of its targets) (Sup. Fig. 4J). Among the enormous sets of putative targets^81^, this subset may be more likely to be valid in the context(s) modeled by differentiating gastruloids.

### Quantitative phosphoproteomics reveals kinase activities across gastruloid development

**Figure 5.**
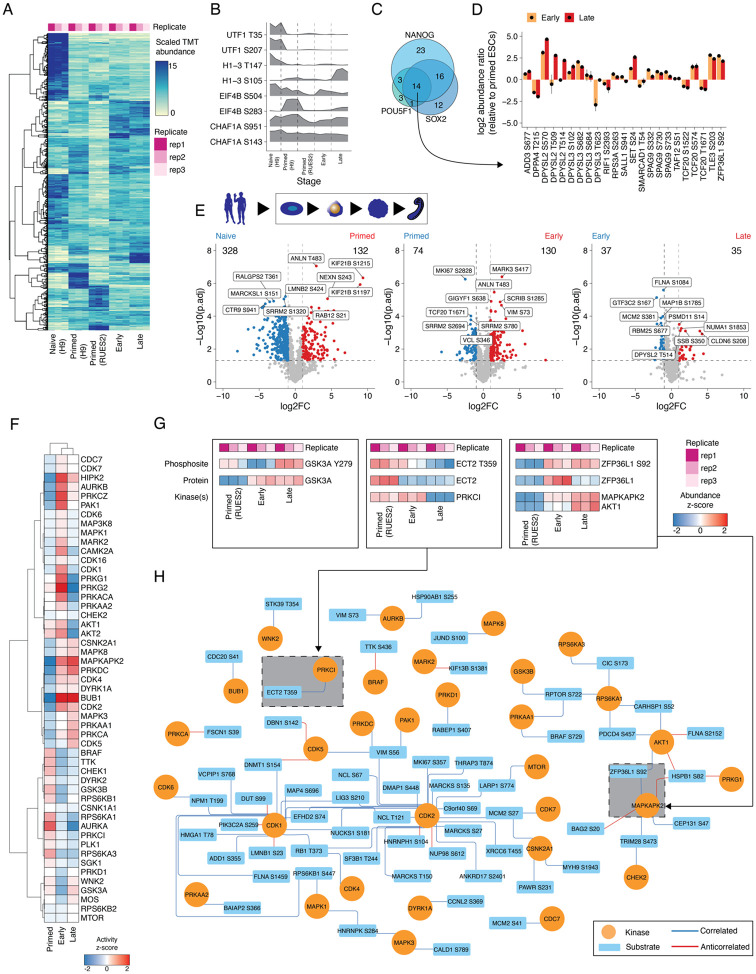
Quantitative phosphoproteomics reveals kinase activities across gastruloid development. **(A)** The temporal dynamics of phosphorylated peptides across human gastruloid development. Rows indicate phosphosites, while columns signify sample type. Color scale indicates the scaled TMT abundance of individual phosphopeptides. **(B)** Ridgeplots depicting the characteristic phosphorylation states within a given protein. **(C)** Venn diagrams depicting the detection of phosphorylated proteins that are targets of pluripotency factors SOX2, POU5F1 and NANOG. Gene sets curated from Van Hoof et al.^110^ **(D)** Phosphosites associated with downstream targets of pluripotency factors. Y-axis indicates the log_2_ abundance ratio of early (yellow) or late (red) gastruloids to primed RUES2-GLR ESCs. Mean abundance ratios are indicated with dots and error bars represent the standard deviation. **(E)** Volcano plots of phosphosite abundance changes across consecutive stages of human gastruloid developmental stages. X-axis represents the log_2_ fold change between 2 timepoints and the y-axis represents the negative log10 of the BH-adjusted p-value. Phosphosites were normalized to their protein levels before differential expression testing. Labeled points indicate the top 5 most significant differentially expressed phosphosites between pairs of comparisons. **(F)** Heatmap depicting the z-scores of kinase-substrate enrichment analysis. **(G)** Representative examples of temporal phosphosite dynamics in comparison to their respective proteins and cognate kinases. Color scale indicates the abundance z-score. ECT2 T359 was correlated with PRKCI, while ZFP36L1 S92 was strongly correlated with both MAPKAPK2 and AKT1. **(H)** Network of kinases (circles) connecting to their substrates (rectangles). Pairs annotated from PhosphositePlus. Edge colors indicate correlated (blue) or anticorrelated (red) relationships (absolute r_Pearson_ >= 0.5) between kinase and substrate phosphosite nodes.

Developmental programs are largely driven by core signaling pathways (*e.g.* WNT, BMP, FGF) that are dynamically regulated via post-translational modifications (PTMs)^25^. To identify PTMs that might underlie such regulation during gastruloid development, we applied time-resolved quantitative phosphoproteomics to our sample set (Fig. 1A-B; Fig. 5A; Sup. Fig. 5A-B; Sup. Table 10). Human and mouse phosphosites were well correlated with their protein abundances (median r_Pearson_ = 0.71 (human) and 0.84 (mouse)) and included residues of known stem cell markers such as DOT1L, LIN28A, SALL1, and UTF1 (Sup. Fig. 5C-D). In addition to temporal trends in individual phosphosites across gastruloid development, we also identified patterns of sharing or difference for phosphosites in the same protein. For example, multiple UTF1 phosphosites were enriched in naïve ESCs^38,82^, while for H1-3, T147 phosphorylation abundance was highest in naïve ESCs while S105 phosphorylation abundance was highest in fully differentiated gastruloids (Fig. 5B).

We reasoned that phosphoproteomics could discern the abundance and state of proteins targeted by the chemical treatments used to induce gastruloid development. In particular, mouse gastruloids were treated with Chiron, a small molecule used to activate WNT signaling by inhibiting GSK3 kinase activity^83-85^. We found that Gsk3a and Gsk3b protein abundances were elevated in late gastruloids (after Chiron had been removed) and both kinases were anticorrelated to WNT signaling effectors Ctnna1 and Ctnnb1. Interestingly, we observed that the abundance of Gsk3a-activating phosphorylation at Y279 was inversely correlated with Chiron treatment, potentially reflecting Chiron-dependent perturbation of Gsk3a activity during mouse gastruloid induction (Sup. Fig. 5E). This observation highlights the potential impact of Chiron on GSK3 phosphorylation states and is consistent with a recent study predicting decreased Gsk3 activity in 72 hr mouse gastruloids compared to ESCs^86^.

We mapped phosphosites in our datasets onto known targets of pluripotency markers POU5F1, SOX2 and NANOG curated from previous studies^39,40^ (Sup. Table 11). We identified a total of 113 phosphosites on 72 proteins downstream of these pluripotency markers and focused on the subset that we successfully measured with proteomics. Of these, 14 proteins were shared targets of the pluripotency markers POU5F1, SOX2, and NANOG and phosphorylation sites on these 14 targets exhibited temporal changes in total phosphorylation over the course of gastruloid development (Fig. 5C). For example, compared to naïve ESCs, DPPA4 phosphorylation (T215) was more abundant in primed ESCs; however, we found residues on DPYSL2 (S570 and T514) and DPYSL3 (S682 and S684) tended to have more total phosphorylation in early and late gastruloids. These results are consistent with previous work as DPPA4 is a known marker of pluripotency^87^ while DYSL2 and DPYSL3 are associated with nervous system development^88^. TCF20, a transcriptional coactivator associated with neurodevelopmental disorders, displayed two distinct patterns with respect to its detected phosphosites. TCF20 residues S1522 and S1671 had maximal phosphosite abundance in primed ESCs, correlating with upstream pluripotency factors NANOG, POU5F, and SOX2. However, phosphorylation of TCF20 S574, however, was most abundant in early and late gastruloids, when pluripotency factor abundance is low (Fig. 5D). Consistent with previous work^39,40^, these data suggest divergent post-translational control of developmental transcription factors, possibly modulated by canonical pluripotency factors.

We next sought to assess conservation of cellular signaling axes in human vs. mouse gastruloid development. Using matched developmental timepoints between humans and mice (primed ESCs, early and late gastruloids), we calculated the correlation between orthologous phosphosite residues. We observed a wide distribution of Pearson correlation coefficients (median r_Pearson_ = −0.14) suggesting divergent phosphorylation dynamics between both species (Sup. Fig. 5F). The neural stem cell regulator DPYSL2 T514/Dpysl2 T514 phosphosite abundances were consistent moving from mouse to human gastruloids, while chaperone HSP90AB1 S255/Hsp90ab1 S255 and ribosomal protein kinase RPS6KB1 S447/Rps6kb1 S447 displayed species-specific phosphosite dynamics (Sup. Fig. 5F). Conserved phosphorylation sites for primed stem cell marker DNMT3B S100 and Dnmt3b S116 exhibited highly consistent phosphorylation profiles across gastruloid differentiation. Notably, the N-terminal region around the S100/S116 site has been challenging to resolve in structural studies^89^, lies outside of the methyltransferase catalytic domain of DNMT3B, and is important for DNA binding^89,90^. Owing to DNA methylation’s established role in mouse epiblast^91-93^ and primed ESCs^94^, the conserved phosphorylation dynamics of DNMT3B S100 and Dnmt3b S116 suggests the possibility of posttranslational control of DNA methylation via these sites in early development.

We next sought to map differential phosphorylation changes across stages of gastruloid development. Given our previous observation of high correlation between proteins and their phosphosites, we normalized the phosphosite changes to their corresponding protein fold changes to map protein-level-independent abundance changes in phosphorylation. While these reduced the numbers of differentially expressed phosphosites, we still identified hundreds of sites changing independently of the protein abundances (Fig. 5E; Sup. Fig. 5G). The highest number of differential phosphosites were detected between naïve and primed H9 ESCs while the lowest number of differential phosphosites was in comparing early and late gastruloids.

Towards nominating driver kinases for temporally dynamic phosphorylation states, we identified kinases detected in our data based on established kinome databases^95^. We detected 262 of 534 (49%) known human kinases spanning all kinase classes (Sup. Fig. 5H). We performed kinase-substrate enrichment analysis^96-99^ to identify upstream kinase modules associated with phosphorylation across gastruloid development. Upon clustering kinases with similar z-score profiles, we broadly observed three clades of kinase activity (Fig. 5F). In line with a recent study profiling mouse gastruloids^86^, the activities of GSK3B and DYRK2 tended to be higher in primed ESCs compared to human gastruloids. This is expected because like mouse gastruloids, early human gastruloids were also cultured in the presence of the GSK3A/B inhibitor Chiron. The decreased predicted kinase activity of GSK3B in gastruloids is consistent with increased inhibitory N-terminal phosphorylation of GSK3B^100^. We also observed increased phosphorylation of a GSK3A/B peptide (GEPNVSY#ICSR) with exact sequence identity to activating phosphorylation sites^101-103^ of GSK3A (Y279) and GSK3B (Y216). While these sites cannot be disambiguated owing to their tryptic peptide sequences, GSK3B-specific N-terminal phospho-inhibition^103,104^ coupled with increased phosphorylation of the GSK3A/B activating site and a ~1.5-fold higher Chiron IC_50_ for GSK3A (10.1nM for GSK3A; 6.7nM for GSK3B)^105^ hints at differential roles for these isozymes in gastruloid development, consistent with previous findings for the role of GSK3A in ESC differentiation^106^ and nervous system development^107^. Notably, GSK3-activating phosphorylation was modulated with the Chiron treatment (Sup. Fig. 1A) and elevated in late gastruloids (Fig. 5F) when we would expect neural mesodermal progenitor cells to be present^10^. Owing to the poor correlation between GSK3A phosphorylation and protein abundance, these data suggest both coordinated temporal expression and activating phosphorylation of this key signaling axis during gastruloid formation.

Our phosphoproteomics analysis also captured known kinase-substrate relationships including MAPKAPK2 phosphorylation of ZFP36L1 at Ser92 and PRKCI phosphorylation of ECT2 Thr359 (Fig. 5G). ZFP36L1 is a downstream target of NANOG and its protein abundance peaked in early gastruloids, suggestive of post-transcriptional and/or post-translational regulation (Fig. 5D). ZFP36L1 is an RNA binding protein and mediates degradation of transcripts when activated by MAPK signaling. We found that ZFP36L1 Ser92 phosphorylation was correlated with MAPKAPK2’s predicted activity (Fig. 5F,G). At the protein level, NANOG abundance had an inverse relationship with that of ZFP36L1. This finding is in line with previous studies in mESCs^108,109^ and suggests ZFP36L1 Ser92 may play a role in stabilizing ZFP36L1 levels and be involved in the degradation of NANOG during gastruloid development. While AKT1 was known to phosphorylate ZFP36L1 on Ser92, we found that AKT1 activity was predicted to be lower in the late gastruloids, consistent with a temporal association between ZFP36L1 and MAPKAPK2. Overall, over the course of gastruloid development, we observed a median Pearson’s correlation of 0.35 between kinase protein abundances and kinase substrate phosphorylation abundance (Sup. Fig. 5I). There were 62 correlated pairs and 24 anticorrelated pairs with an absolute r_Pearson_ >=0.5, including major kinase classes and many known kinase-substrate relationships including CDK1 substrates RB1, NUCKS1, and LIG3 (Fig. 5H). Taken together, our findings nominate kinase-substrate relationships across gastruloid development, together with their temporal dynamics.

### Co-regulatory networks of protein dynamics in gastruloids link to shared phenotypes and developmental disorders

**Figure 6.**
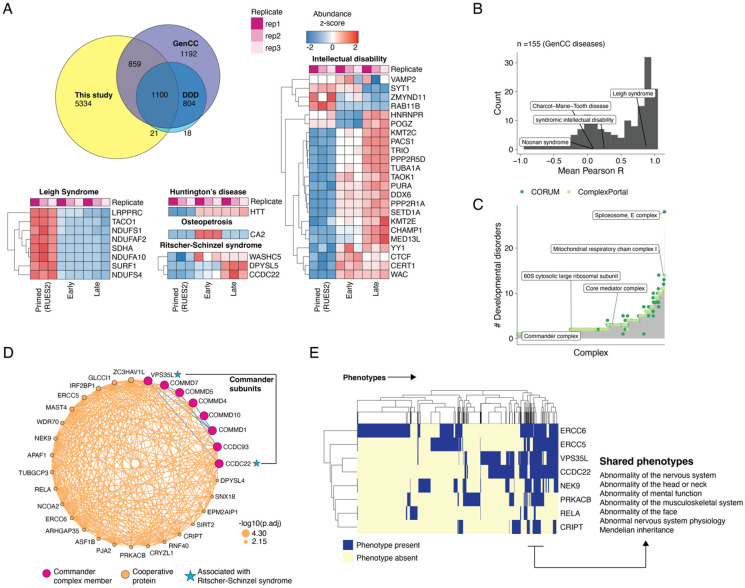
Co-regulatory networks of protein dynamics in gastruloids link to shared phenotypes and developmental disorders. **(A)** Overlap of proteins detected across our dataset, GenCC and DDD. Heatmaps correspond to the temporal abundance changes of human proteins (rows) associated with a specific developmental disorder across human gastruloid development (columns). **(B)** Distribution of mean Pearson correlation coefficients across GenCC disease sets. Only diseases with >=2 genes are plotted. Mean r_Pearson_ was calculated by averaging Pearson correlation coefficients detected pairs of proteins in our dataset. **(C)** Histogram of the number of developmental disorders associated (y-axis) with genes comprising protein complexes (x-axis). **(D)** Co-regulation network of the Commander complex subunits. Size of orange nodes indicates the significance of cooperative association (−log_10_ of the adjusted p-value; Fisher’s exact test, see Methods). Proteins associated with developmental disorders (blue stars) were linked to the Commander complex co-regulation network. **(E)** Heatmap depicting the extent of shared phenotypic overlap (columns) across genes (rows) in the Commander subnetwork.

*In vitro* models of early development offer tractable platforms to model congenital disease states and map the molecular mechanisms underlying them. To investigate the temporal dynamics of proteins linked to developmental disorders, we intersected our dataset with the Gene Curation Coalition (GenCC)^112^ and Deciphering Developmental Disorders (DDD)^113^ databases. There were 1,980 proteins (27%) quantified in our datasets with at least one disease association in at least one of these databases (Fig. 6A; Sup. Table 12). Anecdotally, genes linked to the same disease tended to be co-regulated across gastruloid development. For example, genes associated with Leigh Syndrome, a congenital early-onset neurological disorder associated with mitochondrial dysfunction, tended to be upregulated in primed ESCs, while genes linked with broad intellectual disability mostly showed increased abundance during the gastruloid stages (Fig. 6A). To ask if co-regulation was a general trend among genes associated with the same disease, we calculated the mean Pearson correlation coefficient across all pairs of detected proteins associated with a given disease by GenCC, and found these tended to be positively correlated (average r_Pearson_ = 0.46, Fig. 6B).

Mapping disease-associated genes onto known protein complexes can inform their molecular roles in developmental disorders. There are 461 developmental disease-associated genes whose protein products contribute to 217 ComplexPortal and 631 CORUM complexes (Sup. Table 12). Complexes were associated with an average of 2.95 ± 2.68 developmental diseases, with the spliceosome E complex and mitochondrial respiratory Complex I are associated with the largest number of developmental disorders (Fig. 6C). With the goal of identifying additional disease genes that might be related to these protein complexes, we leveraged our data together with the aforedescribed co-regulatory analysis heuristic (Fig. 3A; Sup. Fig. 3J). Towards supporting these protein-complex associations, we mined BioPlex and BioGrid, which identified 232 edges linking cooperative disease proteins to CORUM complexes, and 180 edges linking cooperative disease proteins to ComplexPortal complexes (Sup. Fig. 6A).

Functional proteomics can be a powerful approach to nominate molecular functions for disease-associated genes with molecular functions, as well as to advance our mechanistic understanding of how their aberrant function in specific developmental contexts might give rise to specific disease phenotypes^114,115^. To illustrate how the data reported here might be useful in this regard, we highlight examples involving Leigh Syndrome and Ritscher-Schinzel Syndrome.

Leigh syndrome is an early-onset mitochondrial neurometabolic disorder impacting the central nervous system. Its symptoms include, ataxia, developmental delay and hypotonia^116^. We found that the protein levels of 51 Leigh Syndrome-associated genes detected in our data were highly correlated with one another in our data (Fig. 6B, mean r_Pearson_ = 0.87). In our co-regulation network, Leigh syndrome proteins clustered with genes associated with central metabolism (*e.g.* Complex I and mitochondrial ATP synthase) and were significantly enriched in a oxidative phosphorylation co-regulation subnetwork (p < 9.6x10^−15^, Fisher’s Exact test, Sup. Fig. 6B).

Ritscher-Schinzel syndrome is a developmental disorder characterized by abnormal craniofacial, cerebellar, and cardiovascular malformations, classically associated with WASHC5 and CCDC22 but more recently with VPS35L and DPYSL5 being implicated as well^117-122^. These four proteins were positively correlated in our data (mean r_Pearson_= 0.78) with two of them (CCDC22 and VPS35L) clustering within a co-regulation network involving subunits of the Commander complex (Fig. 6C). The Commander complex consists of two subcomplexes, CCC and Retriever^123^, and is mainly involved in trafficking of cargo including endosomal recycling of proteins^124^. While we detected all 16 Commander subunits, including the three heterotrimeric components of the Retriever complex (Sup. Fig. 6C), our co-regulation network contained 7 CCC subunits, 1 Retriever subunit, and 23 cooperative proteins (Fig. 6D).

While we detected 2 of the 4 genes associated with Ritscher-Schinzel syndrome, we also observed 7 of the 31 proteins in the Commander network had GenCC disease associations. We hypothesized that cooperative disease-associated proteins in the Commander network would share similar phenotypic features. To measure the extent of phenotypic overlap among disease-associated proteins in the Commander network, we leveraged the Monarch database of gene-phenotype relationships^125^. We found phenotype associations for 8 proteins in the Commander network, which clustered into groups based on shared sub-phenotypes (Fig. 6E). For example, and unsurprisingly given how the syndrome is defined, Ritscher-Schinzel syndrome genes CCDC22 and VPS35L shared highly similar phenotypes. More broadly however, Commander-cooperative proteins exhibited overlapping phenotypic characteristics, including abnormality of the nervous system, mental function, and musculoskeletal system (Fig. 6E). This analysis suggests putative disease associations in genes that may be associated with Leigh syndrome and Ritscher-Schinzel syndrome and offers starting points to characterize disease genes in these developmental contexts.

Finally, to investigate phosphorylation dynamics of disease genes, we intersected our phosphorylation dataset with the DDD database, and found 202 phosphosites across 105 proteins that are temporally quantified in our dataset (Sup. Fig. 6C). For example, phosphosites on CDK13, TCF20 and NONO, genes that are known to be strongly associated with intellectual disability, exhibit varying temporal profiles (Sup. Fig. 6D). For example, TCF20 Ser1522 and Thr1671 peak in primed ESCs while Ser574, similar to its protein levels, peaks during gastruloid differentiation. Thus, our data may serve as a resource for nominating PTM targets for future investigation of the complex regulation of genes underlying congenital and developmental disorders.

## DISCUSSION

In this study, we leveraged the tractable and scalable properties of stem cell derived human and mouse gastruloids to systematically profile temporal changes across four key stages of their development. While the numbers of *in vitro* models of embryogenesis continue to expand and are increasingly characterized with scRNA-seq or scATAC-seq, only recently have they been subjected to phenotyping at the protein level. For example, a recent study applied mass spectrometry to map the temporal protein dynamics across stages of mouse gastruloid development, yielding insights into germ layer proteomes and associated phosphorylation states^86^. However, this study was restricted to mice and stages surrounding gastruloid induction. Thus, we sought to extend the application of these approaches to a human model of gastrulation to enable multi-species comparisons and explore additional developmental states mapping to pre- and post-implantation to gain a more comprehensive view of mammalian gastrulation.

By applying high-throughput quantitative proteomics to both mouse and human gastruloid development, we aimed to generate a dataset that foundationally informed the transcriptional, proteomic and phosphoproteomic dynamics of this model. These differences tended to correspond to systemic shits in biological processes across stages. Notably, we observe that over human gastruloid development, proteins in the TCA cycle tend to be upregulated in primed ESCs as compared to late gastruloids, and late gastruloids display elevated levels of glycolytic proteins. These results suggest a refocusing of cellular composition on metabolic energy production in support of larger organismal changes, in broad agreement with previous studies demonstrating the metabolic shift to glycolysis in post-implantation embryos^126,127^. It is important to note that while previous studies using transcriptomic approaches suggest a bivalent metabolic state in epiblast cells^127^, we found that primed RUES2-GLR cells had increased abundance of oxidative phosphorylation proteins. Additionally, we found that RUES2-GLR proteomes when compared to primed H9 cells displayed an elevated oxidative phosphorylation profile, showing stem cell line-specific metabolic states.

Our data enabled comparison of temporal dynamics of protein expression and conservation (or lack thereof) across human and mouse gastruloid development. While late gastruloids across both species were only modestly correlated, key developmental genes often displayed conserved patterns of expression. For example, in both species the protein abundance for pluripotency markers POU5F1, NANOG, CDH1 were all lower in late gastruloids compared to their stem cell progenitors. Conversely, ZEB2 (a key protein involved in epithelial-mesenchymal transition^128^), SOX9 (a neural crest marker), CDX2 (a caudal axial stem cell marker), and MEIS1 (a cardiomyocyte marker) protein abundances all increased at the protein level in both human and mouse gastruloids. We found conserved upregulated biological processes include regulation of cell differentiation, organ morphogenesis, heart/muscle development, while conserved downregulated processes include amino acid metabolism and transport. These findings highlight the extent of conservation of gastrulation programs across 90 million years of evolutionary divergence.

Upon comparing the stages sampled across both species, we surprisingly found that the proteomes of primed RUES2-GLR cells were closest to those of the early mouse gastruloids. While this association was largely driven by upregulation of mitochondrial proteins, it is possible that RUES2-GLR cells (thought to be between pre- and post-implantation states) may already be primed towards gastrulation at the protein level. Our results thus also highlight potential species-specific differences in staging, particularly with respect to metabolic and mitochondrial states. However, more work is needed to understand the extent of these effects and to rule out the possibility that these are due to cell line-specific differences that do not reflect *in vivo* effects. We imagine that a more continuous sampling of early differentiation, together with computational staging between species^10^ will resolve this question. When comparing protein abundances to their corresponding transcripts, we observe modest correlation (r_Pearson_ = 0.39) with a clear discordance between mitochondrial proteins and transcripts including proteins underlying key metabolic pathways such as oxidative phosphorylation being anticorrelated. However, this was not the case for other pathways such as WNT signaling, steroid biosynthesis and glycolysis. Our findings are in broad agreement with other studies mapping RNA-protein relationships in developmental contexts across a range of organisms^16,18-22^ and highlight the roles of post-transcriptional regulation in gastruloid development as well as the need to study multiple layers of biomolecular composition of organisms during development. Future studies, applying ribosome profiling^129^ and single cell multi-omics of the proteome and transcriptome^130^ may inform the translation and turnover rates of specific proteins across developmental stages.

Studies of co-regulation of protein systems (*i.e.* complexes and pathways) during gastrulation have been limited owing to the lack of tractable and scalable models of mammalian gastrulation. Here we recover hundreds of known macromolecular protein complexes and biochemical pathways and map their dynamics during gastruloid development. In mining our co-regulatory networks of protein expression, we identify thousands of new cooperative proteins associating with existing complexes and pathways suggesting possible developmental roles in gastrulation. While many proteins were discrete to the complexes they cooperated with, we also found shared sets of cooperative proteins. For example, chromatin remodelers (SWI/SNFs and BAFs) and histone methyltransferases (SIN3A/SIN3B histone methyltransferases) and acetyltransferases (HBO complexes) shared cooperative proteins. These relationships represent a resource for further exploration using biochemical assays to disentangle those proteins that directly interact from those in shared pathways.

Finally, we found that co-regulation and network analysis were able to identify disease neighborhoods. Often we observed that genes associated with the same developmental disorders were highly correlated with one other at the protein level. Subunits of the Commander complex are associated with Ritscher-Schinzel syndrome^131,132^. The cooperative network containing this complex consisted of 2 of the 4 genes strongly linked to Ritscher-Schinzel syndrome. Other proteins in the Commander network are associated with similar phenotypic characteristics, potentially for shared reasons, which may serve as a starting point to understanding their roles in gastrulation. Finally, this study offers scalable and multidimensional approaches to provide high-content phenotyping going beyond ubiquitous nucleic acid-centric assays. For example, generalizable protein-focused approaches can be extended to phenotype genetically and chemically perturbed gastruloids along with other clinically relevant stem cell models that are increasingly being used to model embryogenesis^5^.

### Limitations of the study

Though this study quantitatively profiles the transcriptome, proteome, and phosphoproteome across gastruloid development, it is not complete nor comprehensive. First, while we sample 4 stages of embryonic development in gastruloids, profiling more time points with finer windows across gastruloid development would provide greater resolution and enhance our understanding of the temporal dynamics present in these samples. Secondly, though we quantified ~7,500 human and ~8,700 mouse proteins, which represents a substantial portion of the observable proteome^133^, additional coverage of low abundance, developmentally associated genes could be attained using targeted, quantitative mass spectrometric analyses. Thirdly, gastruloids consist of diverse cell types arising from all three germ layers. Our approaches were all bulk measurements and lacked cell-type-specific resolution. Additional characterization of separate cell types with fluorescence activated cell sorting (FACS) before phenotyping mouse gastruloid development with proteomics^86^, could overcome this limitation. Finally, although gastruloids are powerful surrogates to model specific characteristics of early mammalian embryogenesis, they still do not entirely reconstitute embryogenesis *in vitro.* Future multi-omic studies will lay the foundation for advancing these stem cell models to more accurately recapitulate embryogenesis and allow us to better understand the cellular and molecular mechanisms driving embryonic development.

## METHODS

### Mouse cell lines

E14Tg2a cell line was obtained from Dr. Christian Schroeter (Max Planck Institute).

### Mouse naïve ESC culture

Mouse naïve ESCs were maintained in 2iLif medium^84^ containing 3 μM CHIR99021 (Millipore-Sigma, SML1046), 1 μM PD0325901 (Stemcell Technologies, 72184), and 1,000 U/ml LIF (Millipore, ESG1107) and passaged with TrypLE (Thermo, 12604021) every other day onto new wells, which were coated with 0.01% poly-L ornithine (Millipore Sigma, P3655-10MG) and 300 ng/ml Laminin (Corning, 354232).

### Mouse EpiLC differentiation

Mouse EpiLC differentiation was performed as previously described^134^. Briefly, 1x10^5^ mouse naïve ESCs were seeded onto a well on a 12-well plate, which was coated with human plasma fibronectin (Thermo, 33016015) in EpiLC differentiation medium (N2B27 + 20 ng/ml ActivinA + 12 ng/ml bFGF + 1% KSR). The medium was changed a day after the seeding. Day2 EpiLCs were dissociated with TrypLE (Thermo, 12604021) and sampled.

### Mouse gastruloid induction

Mouse gastruloid induction was performed as previously described^9^. Briefly, mESCs cultured in 2iLiF medium were dissociated with TrypLE, and 300 cells were seeded into U-bottomed, non-adherent 96-well plates in N2B27 medium and kept for 48 hours in 37°C 5% CO_2_ incubator. After 48 hours, 150 μl of N2B27 containing 3 μM CHIR99021 was added to each well. At 72 and 96 hours, 150 μl medium was replaced with fresh N2B27 medium lacking CHIR99021. Mouse gastruloids were sampled at 72 and 144 hours after induction.

### Human cell lines

Pluripotent stem cell lines, hESCs (RUES2-GLR), were gifted by Dr. Ali Brivanlou (Rockefeller University). Chemically reset (cR) H9 naïve and primed cells were kindly gifted by Dr. Austin Smith (University of Exeter).

### Human naïve ESC culture

Chemically reset (cR) H9 naïve hESCs were propagated in N2B27 with PXGL (P-1mM PD0325901, 2mM X- XAV939 , G- 2mM Gö 6983 and L- 10 ng/mL L-human LIF) on irradiated MEF feeders as described previously^32,135^. Y-27632 and Geltrex (0.5mL per cm^2^ surface area; Thermo Fisher Scientific, A1413302,) were added during re-plating. To remove MEF cells, cells were passaged on geltrex-coated wells with the 1 μL/cm^2^ and were repeatedly passaged by dissociation with Accutase (Biolegend, 423201) every 3-5 days for five successive passages.

### Human primed ESC culture

Human primed ESCs were cultured in StemFlex (Thermo, A3349401) on Geltrex (Thermo, A1413201) and were routinely passaged using StemPro Accutase (Thermo, A1110501) to new Geltrex-coated wells as recommended by the manufacturer. For the first 24 hrs after passaging, hESCs were cultured in StemFlex with 10 μM of Rho Kinase inhibitor Y-27632 (Sellek, S1049) to prevent apoptosis.

### Human RA-gastruloid induction

Human RA-gastruloids were induced as described previously^10^. Briefly, ~4x10^4 hESCs were plated onto a single well of a Vitronectin-coated 12-well dish (Gibco, A14700) in Nutristem hPSC XF medium (Biological Industries, 05-100-1A) in the presence of 10 μM Y-27632. After 24 hours, the medium was replaced with Nutristem containing 5 μM Y-27632. At 48hrs the medium was replaced with Nutristem containing 4 μM CHIR (Millipore, SML1046). At 72 hrs, the medium was replaced with Nutristem containing 4 μM CHIR and 500 nM RA (Millipore Sigma, R2625). Pre-treated cells were detached using StemPro Accutase, dissociated into single cells suspension, and then 4,000 cells per well of a U-bottom shaped 96-well plate with 50 μl Essential 6 medium (Thermo, A1516401) containing 1 μM CHIR and 5 μM Y-27632. At 24 hrs, 150 μl of Essential 6 medium was added to each well. At 48 hrs, 150 μl of the medium was removed with a multi-channel pipette, and 150 μl of Essential 6 medium containing 5% Matrigel and 100 nM RA was added and maintained at 37°C and 5% CO_2_ until 120 hrs. Human gastruloids were sampled at 24 and 120 hours after induction.

### RNA-seq analysis

#### Sample preparation

Each stage consisted of 2 biological replicates. Approximately 0.5 million cells per replicate were harvested across mouse and human cells across the 4 gastruloid developmental stages. DNA and RNA from each sample were isolated using the Qiagen AllPrep DNA/RNA kit (Qiagen #80204). Approximately, 500ng of total RNA was used as input for library preparation. mRNAs were isolated using the NEBNext Poly(A) mRNA Magnetic Isolation Module (NEB #E7490) and prepared for sequencing using the NEBNext UltraII RNA Library Prep Kit for Illumina (NEB #E7770).

#### Sequencing and data analysis

Concentrations of cDNA libraries across all samples were estimated from either the Qubit (Invitrogen) and/or visualized by Tapestation (Agilent) to ensure standard ranges for library sizes. All libraries were dual-indexed with 8 nucleotide indexes using NEBNext^®^ Multiplex Oligos for Illumina^®^ (Index Primers Set 1) and were sequenced on NextSeq 2000 (Illumina) either by 2x150bp or 2x50bp configuration.

Basecall files were converted to fastq formats using bcl2fastq (Illumina) and demultiplexed on the i5 and i7 indexes. Fastqc was performed to estimate the quality of the reads. Adapter trimming and filtering for low quality reads was performed using Trimmomatic v0.39^136^ either in paired-end or single end mode trimming low-quality reads (<2) at the ends and applying a 4 base sliding window across reads retaining reads with average quality above 15. Depending on the species, trimmed reads were then aligned using STAR^137^ to either the human GRCh38 or mouse GRCm39 reference assemblies. Human samples had an average unique mapping rate of 64% while those of mouse samples were 48%. Finally, count matrices for each species were then generated with bam files using FeatureCounts.

### Mass spectrometry data collection

#### Sample preparation

For each stage analyzed we collected 1-2.5 million cells per replicate across 4 gastruloid developmental stages. Stem cells across each stage were harvested from culture plates by enzymatic dissociation using Accutase^™^ (StemCell Technologies, #07920). Since each gastruloid is cultured in a single well of a 96-well U-bottom plate, gastruloids were first pooled together to reach the 2.5 million cell number and gently centrifuged at 500g for 5 min to remove growth media followed by Accutase treatment to dissociate the gastruloids. Once dissociated, Accutase treatment for both gastruloid and stem cell samples was quenched by addition of a wash buffer consisting of either StemFlex or mTeSR+ along with rock inhibitor (Y-27632). Finally cells were washed twice with PBS to remove cell debris, lysed cells, and matrigel from the samples. Samples were finally stored at −80C after aspirating the PBS before proceeding to protein isolation.

Cell pellets were thawed on ice and resuspended in lysis buffer (8M urea, 250mM EPPS pH 8.5, 50 mM NaCl, Roche protease inhibitor cocktail, Roche PhosSTOP). The cell pellets were homogenized using a 21-gauge needle to syringe pump lysate. Lysates were cleared by centrifugation at 21,130 g at 4°C for 30 minutes. Supernatants were placed in clean microcentrifuge tubes and a BCA assay (Pierce) was performed to determine protein concentrations. Lysate containing 25 ug of protein material for biological triplicates at each point of gastrulation were reduced and alkylated with 5 mM Dithiothreitol (DTT) for 30 minutes at room temperature and 20 mM Iodoacetamide (IAA) for 1 hour in the dark at room temperature. The IAA reaction was then quenched with 15 mM DTT. Single-pot solid phase sample preparation (SP3)^138^ using Sera-Mag SpeedBeads was performed to desalt the reduced and alkylated samples. An on-bead protein digestion was performed by adding LysC at a 1:100 ratio (protease:protein) overnight (16-24 hours) on a thermocycler at room temperature then adding trypsin at a 1:100 ratio for 6 hours at 37°C at 900 rpm. TMTpro was used to label each sample at a 2.5:1 ratio of TMTpro reagents to the peptide mixtures for each sample. Samples were left at room temperature for 1 hour for TMTpro labeling and labeling efficiency was verified to be >99% for lysines and >97% for N-termini. The labeling reaction was quenched with 5% hydroxylamine diluted to a concentration of 0.3% for 15 minutes at room temperature. Samples were then placed on a magnetic rack to aggregate SP3 beads and labeled peptide supernatants from each sample were pooled. The pooled sample was then partially dried down by speed-vac and 10% formic acid was added to bring the pH of the pooled sample to below 3 for desalting. The pooled sample was desalted using a Sep-Pak C18 cartridge (Waters) and then dried down completely.

#### Phosphoproteomics sample preparation

Pooled sample was resuspended in 94 uL of 80% acetonitrile and 0.1% trifluoroacetic acid for Fe^3+^-NTA magnetic bead phosphopeptide enrichment^139^. 100 uL of 75% acetonitrile 10% formic acid was added to a clean microcentrifuge tube and the Fe^3+^-NTA magnetic beads were washed twice with 1 mL of 80% acetonitrile and 0.1% trifluoroacetic acid and the supernatant was removed. After the final wash, the peptides in 94 uL of 80% acetonitrile and 0.1% trifluoroacetic acid were added to the tube with the washed beads. The sample was vortexed and incubated for 30 min on thermoshaker (250 rpm, 25 C). After the incubation period, the sample was washed 3 times with 200 uL of 80% acetonitrile and 0.1% trifluoroacetic acid and all flowthrough was saved in a clean microcentrifuge tube as it contains non-phosphorylated peptides. 100 uL of 50% acetonitrile and 2.5% NH_4_OH was added to elute phosphorylated peptides from magnetic beads and then sample was transferred to tube with 100 uL of 75% acetonitrile and 10% formic acid. The phosphopeptide enriched sample was dried down immediately by speed-vac and resuspended in 100 uL of 5% formic acid and a C18 stage tip was used to desalt the phosphopeptide enriched sample. The sample was then transferred to a MS insert vial that was placed within a microcentrifuge tube. The sample was placed in a −80 C freezer for 30 minutes and then dried down completely in a speed vacuum. The sample was then resuspended in 10 uL of 2% formic acid and 5% acetonitrile within the MS insert vial.

#### Total proteomics sample preparation

The saved flowthrough was dried down using the speed vacuum, resuspended in 500 uL of 5% formic acid, and a Sep-Pak C18 cartridge (Waters) was used to desalt the sample. The flowthrough sample was dried down completely in speed vacuum after desalting. The flowthrough sample was resuspended and neutralized in 1 mL of 10 mM ammonium bicarbonate/90% acetonitrile and dried down completely in speed vacuum again. Sample was resuspended in 115 uL 10 mM ammonium bicarbonate and 5% acetonitrile and 110 uL were transferred to a sample vial. High-pH Reverse-Phase HPLC Fractionation was performed on the flowthrough sample using an Agilent 1200 HPLC system. After HPLC fractionation^140^, fractions were dried down in speed-vac, resuspended in 100 uL of 5% formic acid, and cleaned via C18 stage tip. Elution from each stage tipped fraction was placed in a MS insert vial and dried down in vial. Fractions were then resuspended in 5 uL of 2% formic acid 5% acetonitrile within the MS insert vial.

### Mass spectrometry data acquisition

#### Proteomics

All analyses were performed using an Orbitrap Eclipse Tribrid Mass Spectrometer (Thermo Fisher Scientific) in-line with an Easy-nLC 1200 autosampler (Thermo Fisher Scientific). The peptides underwent separation using a 15 cm-long C18 column with a 75 μm inner diameter, with a particle size of 1.7 μm (IonOpticks). Each fraction collected from the off-line fractionation was analyzed using a 90 min gradient of 2% to 26% acetonitrile in 0.125% formic acid with a flow rate of 500 nl/min. The MS1 resolution was set to 120,000 with a scan range of 400-2000 m/z, a normalized automatic gain control (AGC) target of 200%, and a maximum injection time of 50 ms. The FAIMS voltage was cycled between activated at a constant compensation voltages (CV) of −40 V, −60, and −80 V. MS2 scans were collected with an AGC target of 200%, maximum injection time of 50 ms, isolation window of 0.5 m/z, CID collision energy of 35% (10ms activation time), and “Rapid” scan rate. SPS-MS3^141^ scans were triggered based on the real-time search (RTS) filter^35^. Briefly, RTS was run by searching species specific Uniprot protein databases (downloaded 04/2023) for mouse (taxid: 10090) and human (taxid: 9606) with static modifications for carbamidomethylation (57.0215) on cysteines and TMTpro acylation (304.2071) on peptide N-termini and lysines; variable modification of oxidation (15.9949) on methionines, one missed cleavage, a maximum of three variable modifications per peptide. Scan parameters of the SPS-MS3 were set to collect data on 10 SPS ions at a resolution of 50,000, AGC target of 400%, maximum injection time of 150 ms, and HCD normalized collision energy of 45%.

#### Phosphoproteomics

Duplicate injections (4 μL) were analyzed on an Orbitrap Eclipse Tribrid Mass Spectrometer (Thermo Fisher Scientific) along with an Easy-nLC 1200 autosampler (Thermo Fisher Scientific). The peptides underwent separation using a 15 cm-long C18 column with a 75 μm inner diameter, with a particle size of 1.7 μm (IonOpticks). Each fraction was analyzed using a 90 min gradient of 2% to 26% acetonitrile in 0.125% formic acid with a flow rate of 400 nl/min. The MS1 scan resolution was set to 120,000 with a scan range of 400-1800 m/z, a normalized AGC target of 200%, and a maximum injection time of 50 ms. The FAIMS voltage was cycled between compensation voltages of −40, −60, and −80 V. MS2 scans were collected with an AGC target of 250%, maximum injection time of 35 ms, isolation window of 0.5 m/z, CID-Multistage Activation (MSA) collision energy of 35% (10ms activation time) with additional activation of the neutral loss mass of n-97.9763, and “Rapid” scan rate. For SPS-MS3 scans^141^ a resolution of 50,000, AGC target of 300%, maximum injection time of 86 ms, and HCD normalized collision energy of 45%.

### Proteomic and phosphoproteomic data analysis

#### Peptide spectral matching

Raw files were searched against the relevant annotated proteome from Uniprot (Human: October 2020; Mouse: March 2021). Sequences of common contaminant proteins and decoy proteins were added to the Uniprot FASTA file to also be searched. Comet search algorithm^142^ was utilized to match peptides to spectra with the following parameters: 20 ppm precursor tolerance, fragment_tolerance of 1.005, TMTpro labels (304.207145) on peptide N-termini and lysine residues, alkylation of cysteine residues (57.0214637236) as static modifications, and methionine oxidation (15.9949146221) as a variable modification. Phosphoproteomics runs were also searched for phosphorylation as a variable modification on serine, threonine, and tyrosine residues (79.9663304104). Peptide-spectrum matches (PSMs) were filtered to a 1% false discovery rate (FDR) using a linear discriminant analysis^35^. Proteins were filtered to an FDR of 1% using the rules of protein parsimony and the protein picker methods^143^. For quantitation, PSMs were required to have a summed TMT reporter ion signal-to-noise ≥100^141^.

#### Protein module analysis

All quantified proteins were mapped onto known transcription factors (curated from the Transcription Factor Database^144^, protein complexes (curated from CORUM^61^ and EMBL ComplexPortal^62^), biochemical pathways (curated from BioCarta^56^, KEGG^57^, PID^59^, Reactome^58^ and WikiPathways^55^), subcellular localization (curated from Human Protein Atlas^29,145^), and Gene Ontology (GO) terms^41^. For biochemical pathways and complexes, we filtered module sets to those where we detected greater than 2 members. With respect to subcellular locations, if a protein in Human Protein Atlas was listed as localized to multiple regions in its main subcellular location, we considered each location as unique. We avoided searching our data against overly broad descriptions of GO terms by filtering for terms containing fewer than or equal to 150 genes and greater than 2 members detected in our data. All mappings were based on Uniprot annotations^69,146^ unless otherwise stated.

#### Correlation network construction and network analysis

We first intersected the human and mouse protein datasets and used 6,261 proteins that were observed across the shared timepoints within a cell line *i.e.* primed ESCs, early and late gastruloids. We normalized each protein’s abundance in given replicate to its respective species geometric mean and log_2_ transformed values for subsequent analysis unless otherwise stated. To construct our correlation network, we first calculated the Pearson correlation coefficients (r_Pearson_) across all 19,596,930 possible pairs of proteins. Since we already calculated r_Pearson_ across all possible pairs of proteins, we permuted sample labels across our dataset to generate the null distribution of correlation coefficients. We then stringently filtered the network edges with Benjamini-Hochberg (BH) adjusted p-values < 0.01 and absolute r_Pearson_ >= 0.95. This step filtered the network down to 489,417 (301,561 correlated and 187,856 anticorrelated) pairs and was used for subsequent network analysis.

#### Edge annotation in correlation network

We considered seven major annotations as literature evidence for any given edge: 1) Protein-protein interaction, 2) Belonging to the same protein complex or 3) biochemical pathway, 4) GO biological process, 5) GO molecular function, 6) GO cellular component or 7) Subcellular location. Protein complex annotations were obtained from CORUM^61^ (downloaded 9/12/2022) and ComplexPortal^62^ (downloaded 1/7/2024). Annotated gene sets for pathways^55-59^ and GO^41^ were downloaded from the Molecular Signatures Database^147^. Protein localization annotations were curated from Human Protein Atlas^29,145^. Networks were illustrated using the igraph R package or Cytoscape^148^.

#### Bioinformatic identification cooperative protein interactions

We searched all nodes in our correlation network against known complexes and pathways which consisted of at least 3 subunits. We adapted a previously described approach^60^ and employed a Fisher’s exact test to compute statistical enrichment of cooperative complexes with established modules. For each protein complex or pathway module, we tested its neighboring proteins (first-degree edges) for significant association with a particular module and termed those as cooperative proteins. For each protein tested, we first counted the number of edges that it shared with the established module, second we counted the number of edges that linked the module to other proteins (excluding the candidate protein) in the network, third we counted the number of edges the candidate protein had to rest of the correlation network (*i.e.* excluding the module of interest) and finally, we counted the number of edges that were not associated with the candidate protein nor the module of interest. These edge counts were used to compute statistical significance using Fisher's exact test. We independently repeated this test for all 6,261 proteins against 1,357 known protein complexes and select metabolic pathways. The p-values obtained were adjusted for multiple hypothesis testing using the BH procedure and only cooperative proteins with adjusted p-value < 0.05 were considered significant.

#### Comparison of RNA and protein abundance analysis

Global RNA-protein correlations were calculated using all 9 observations of transcripts and proteins across mouse and human gastruloid development. To ensure stringent analysis, we filtered for genes detected in both species for the downstream analysis. Pseudocounts of 1 were added to filtered count matrices and were converted to transcripts per million (TPM). Mean transcript and protein abundances were converted to log2 fold change ratios to their respective species geometric mean. For every gene, we calculated the per-gene RNA-protein correlation (r_Pearson_) using a vector of abundances across 9 samples. GO term enrichment of biological processes in correlelated and anticorrelated genes was performed using ClusterProfiler^149^. We intersected the 6010 genes detected across both datasets with Human Protein Atlas^29^ for subcellular locations, CORUM^61^ and ComplexPortal^62^ for protein complexes and KEGG for biochemical pathways^57^. To measure the extent of correlation of transcripts and RNAs within mouse timepoints we calculated the ratio of protein to RNA mean fold changes across each timepoint. In summary, a discordance of 0 implied that the protein and RNAs were highly correlated while discordance less than 0 implied that the RNAs were more abundant than protein levels and vice versa. Discordance scores for protein complexes was calculated by taking the median protein-RNA correlation across constituent members. To prevent averaging pairs of proteins, we only considered complexes where more than 2 proteins were detected in our data. Transcriptional signatures of stage specific mouse transcription factors were detected as follows. First, we calculated the Pearson correlation comparing transcription factor protein abundances to all observed transcripts. We subset the resulting correlation matrix to identify protein-transcript pairs with high correlation (r_Pearson_ >= 0.9) and used TFLink^81^ to select only transcripts that were annotated as targets of specific transcription factors. We confirmed the identified transcription factor targets displayed similar temporal regulation to their upstream transcription factor by comparing target transcript abundance at each stage to determine the maximum transcript abundance.

#### Phosphoprotein and Kinase analysis

For differential expression testing and analysis, in every pairwise comparison, log2 ratios for all quantified phosphosites were calculated following subtraction of the log2 ratios of the corresponding proteins to identify protein independent phosphorylation changes. Kinase substrate pairs were curated from PhosphositePlus^98^. Human kinases were annotated using KinMap^111^. For Kinase-Substrate prediction and enrichment analysis, for each phosphosite, we first calculated the log2 fold change ratio to the row mean (across all samples) subtracted the corresponding protein log2 fold change ratios and used that as input into the KSEA app^96^ with a minimum substrate cutoff >=2 to calculate z-scores for kinases. Kinase substrate pairs with absolute r_Pearson_ >= 0.5 were visualized as a network using Cytoscape^148^.

## Supplementary Material

Supplement 1 Supplementary Table 1- Quantified protein intensities across human and mouse gastruloid development datasets
Supplementary Table 2- Gene Ontology (GO) enrichments of protein clusters with similar temporal profiles
Supplementary Table 3- Pairwise protein correlation network
Supplementary Table 4- Summary statistics of the protein correlation network
Supplementary Table 5- Cooperative proteins of protein complexes
Supplementary Table 6- Jaccard index matrix of cooperative protein pair overlap
Supplementary Table 7- Protein-RNA discordance across the entire dataset
Supplementary Table 8- Protein-RNA correlation across protein complexes and pathways
Supplementary Table 9- Stage-specific GO enrichments for discordant gene sets across mouse gastruloid development
Supplementary Table 10- Quantified phosphosite intensities across human and mouse gastruloid development datasets
Supplementary Table 11- Phosphosites of proteins downstream of pluripotency markers POU5F1, NANOG, and POU5F1
Supplementary Table 12- Disease associated genes and complexes quantified in the human dataset

Supplement 2
