# Supplementary material for: The proteomic landscape and temporal dynamics of mammalian gastruloid development
Source: bioRxiv. 2024 Sep 7:2024.09.05.609098. Preprint. [Version 1] doi: 10.1101/2024.09.05.609098 (PMC11398484; doi:10.1101/2024.09.05.609098)
Supplement: Supplement 2 [file NIHPP2024.09.05.609098v1-supplement-2.pdf]

bioRxiv preprint doi: https://doi.org/10.1101/2024.09.05.609098; this version posted September 7, 2024. The copyright holder for this preprint (which was not certified by peer review) is the author/funder, who has granted bioRxiv a license to display the preprint in perpetuity. It is made available under aCC-BY 4.0 International license.

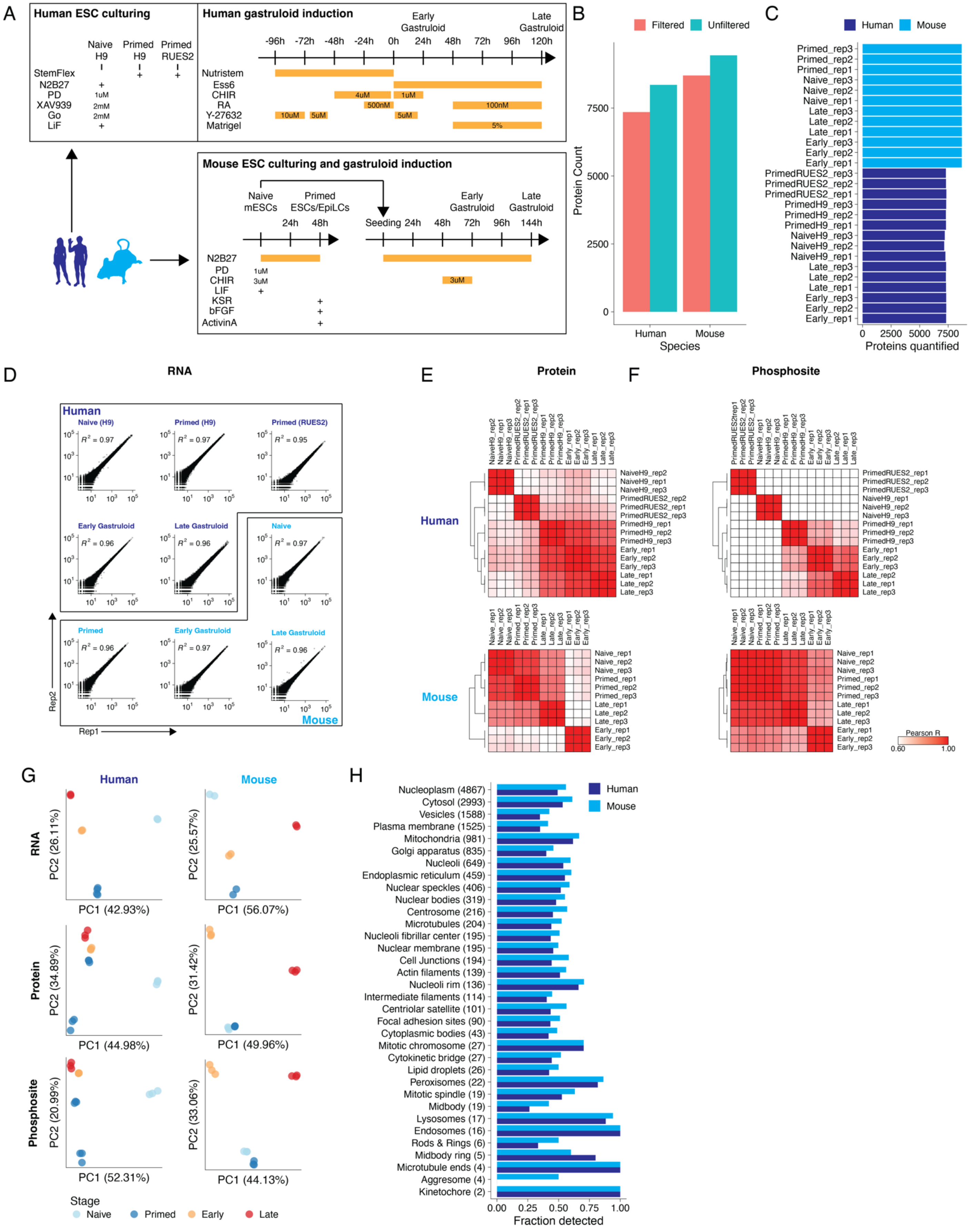

bioRxiv preprint doi: https://doi.org/10.1101/2024.09.05.609098; this version posted September 7, 2024. The copyright holder for this preprint (which was not certified by peer review) is the author/funder, who has granted bioRxiv a license to display the preprint in perpetuity. It is made available under aCC-BY 4.0 International license.

Supplementary Figure 1. Mapping the dynamics of gastruloid development using multi-omics. (A) Timeline and conditions for human and mouse ESC culturing and gastruloid induction. **(B)** Total numbers of proteins quantified across all human or mouse samples. Protein identifications were filtered to a 1% FDR and required summed TMTpro reporter ion signal-to-noise ratios >100 for quantitation. **(C)** Total number of proteins quantified within each sample/replicate. **(D)** Scatterplots comparing the RNA counts between biological replicates for each sample. **(E-F)** All-by-all sample similarity matrices of pairwise Pearson correlation coefficients ($r_{Pearson}$) calculated from summed protein **(E)** or phosphosite intensities **(F)** across human (top) or mouse (bottom) samples. **(G)** PCA plots of PC1 vs. PC2 using RNA (top), protein (middle) or phosphosite (bottom) data across human (left) or mouse (right) samples. **(H)** Fraction of proteins assigned to each of 34 subcellular localizations by the Human Protein Atlas that were successfully detected here. Numbers within brackets indicate the total numbers of proteins within each class shown.

bioRxiv preprint doi: https://doi.org/10.1101/2024.09.05.609098; this version posted September 7, 2024. The copyright holder for this preprint (which was not certified by peer review) is the author/funder, who has granted bioRxiv a license to display the preprint in perpetuity. It is made available under aCC-BY 4.0 International license.

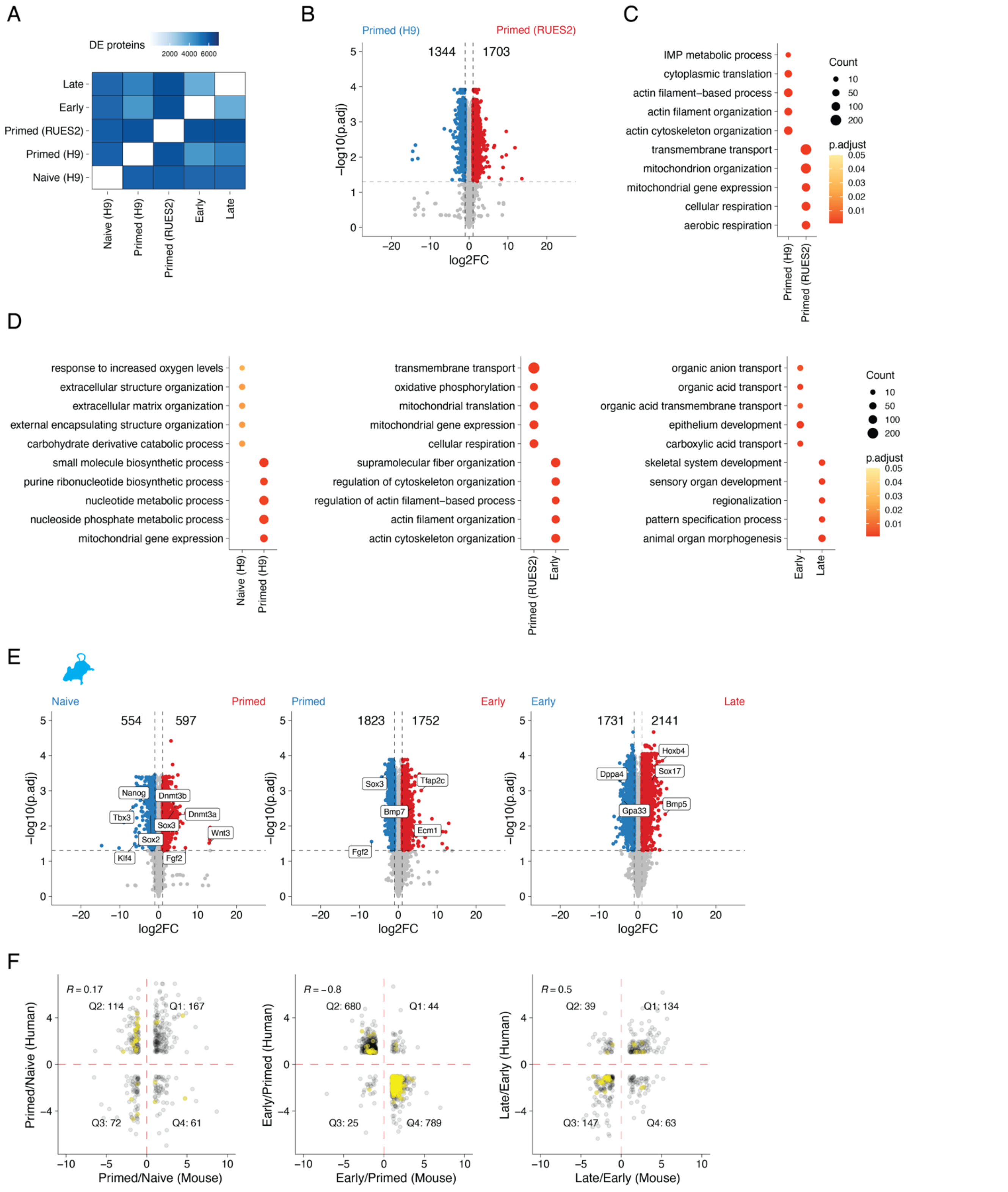

Supplementary Figure 2. Mapping differentially expressed biological processes across gastruloid development. (A) Heatmap indicating the number of differentially expressed proteins (DEPs) between pairs of human samples. DEPs filtered to an absolute $\log_2$ fold change >= 1 and BH-adjusted p-value < 0.05. **(B)** Volcano plot depicting the DEPs between primed H9 vs. primed RUES2-GLR ESCs. **(C)** Dot plot indicating the GO terms enriched in DEPs between primed H9 vs. primed RUES2-GLR ESCs. **(D)** Dot plots indicating the GO terms enriched in DEPs between adjacent stages of human samples. Color scales for dot plots indicate the BH-adjusted p-value and sizes of dots indicate the number of genes detected within each term. **(E)** Volcano plots depicting the DEPs between adjacent stages of mouse

bioRxiv preprint doi: https://doi.org/10.1101/2024.09.05.609098; this version posted September 7, 2024. The copyright holder for this preprint (which was not certified by peer review) is the author/funder, who has granted bioRxiv a license to display the preprint in perpetuity. It is made available under aCC-BY 4.0 International license.

samples, where x-axis represents the $\log_2$ fold change between two adjacent timepoints and y-axis represents the negative $\log_{10}$ of the BH-adjusted p-value. **(F)** Scatter plots comparing mouse and human proteomes across adjacent stages. Comparisons were filtered to proteins with an absolute $\log_2$ fold change >= 1 across both species. Mitochondrial proteins highlighted in yellow.

bioRxiv preprint doi: https://doi.org/10.1101/2024.09.05.609098; this version posted September 7, 2024. The copyright holder for this preprint (which was not certified by peer review) is the author/funder, who has granted bioRxiv a license to display the preprint in perpetuity. It is made available under aCC-BY 4.0 International license.

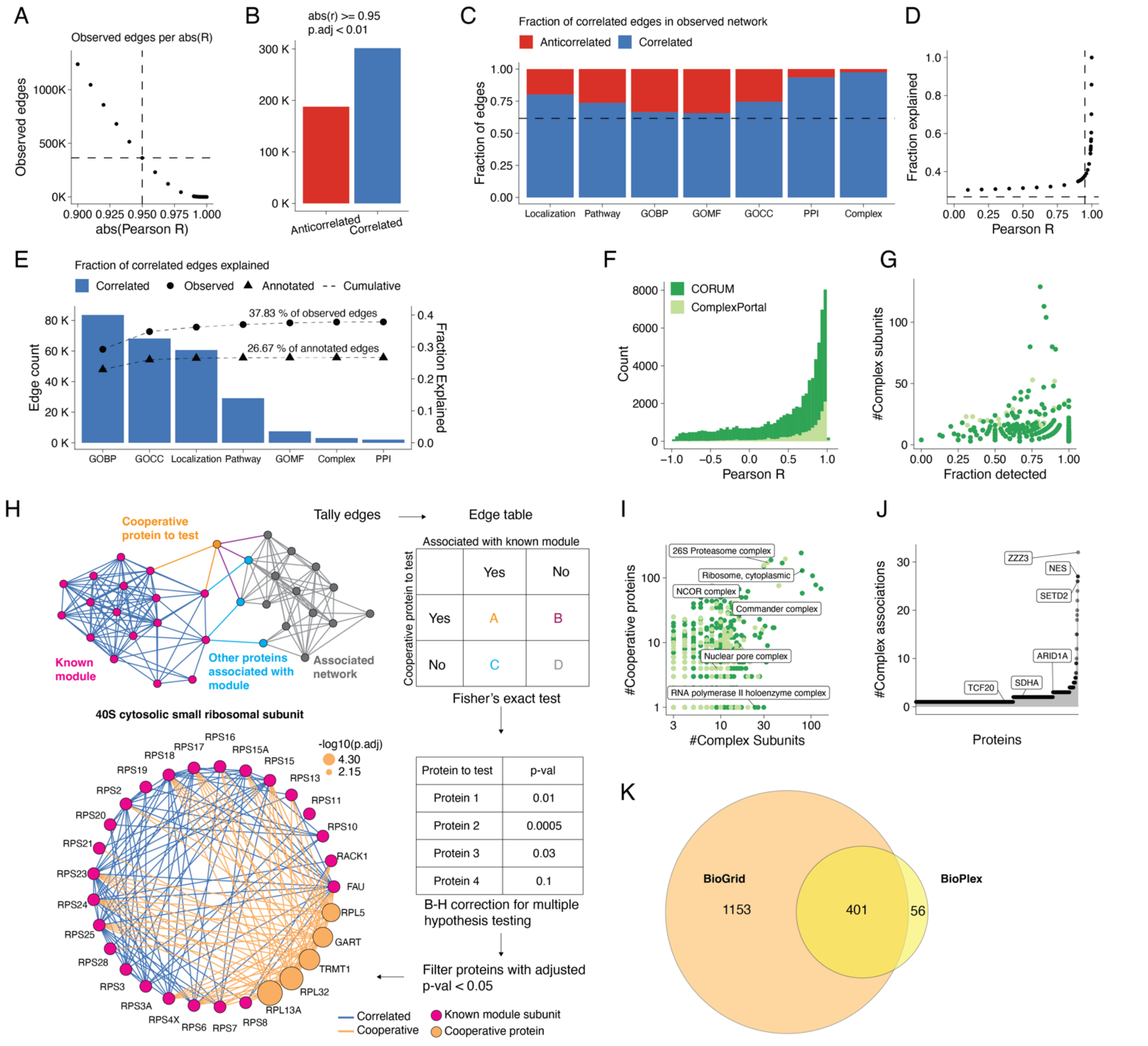

Supplementary Figure 3. Mapping pairwise protein co-regulation onto known protein modules identifies cooperative protein associations across gastruloid development. (A) Number of observed edges (y-axis) in the correlation network as a function of absolute $r_{Pearson}$ (x-axis). **(B)** Summary of correlated and anticorrelated edges in the network. **(C)** Fraction of correlated and anticorrelated edges stratified by GOBP, GOCC, Localization, Pathway, GOMF, BioPlex PPI, and Complex databases. Dashed line indicates the fraction of positively correlated edges in the trimmed network. **(D)** Fraction of correlated edges in the trimmed network explained by at least one database (y-axis) as a function of $r_{Pearson}$ (x-axis). Horizontal and vertical dashed lines respectively indicate the fraction of edges explained in annotated network and $r_{Pearson}$ at 0.95. **(E)** Summary of correlated edges in the trimmed network explained by shared membership in a Gene Ontology biological process (GOBP), cellular component (GOCC), molecular function (GOMF), localization, pathway, protein-protein interaction (BioPlex) or protein complex. Dotted line indicates the cumulative number of edges explained within the observed (circles) and annotated (triangle) networks. **(F)** Distribution of $r_{Pearson}$ for protein pairs in CORUM and ComplexPortal complexes. **(G)** Fraction of complexes detected in correlation network (x-axis) versus size of protein complex (y-axis). Dots colored by database used to curate the protein complexes. **(H)** Workflow to map cooperative proteins associated with detected modules. **(I)** Number of cooperative proteins detected (y-axis) as a function of complex size (x-axis). **(J)** The number of annotated ComplexPortal complexes that were found to be cooperative with each individual protein in the correlation analysis (x-axis), *e.g.* ZZZ3 was assigned as a cooperative protein to 32

bioRxiv preprint doi: https://doi.org/10.1101/2024.09.05.609098; this version posted September 7, 2024. The copyright holder for this preprint (which was not certified by peer review) is the author/funder, who has granted bioRxiv a license to display the preprint in perpetuity. It is made available under aCC-BY 4.0 International license.

ComplexPortal protein complexes. **(K)** Venn diagram indicating the number of cooperative proteins with physical protein-protein interaction evidence to at least one subunit of their associated complex.

bioRxiv preprint doi: https://doi.org/10.1101/2024.09.05.609098; this version posted September 7, 2024. The copyright holder for this preprint (which was not certified by peer review) is the author/funder, who has granted bioRxiv a license to display the preprint in perpetuity. It is made available under aCC-BY 4.0 International license.

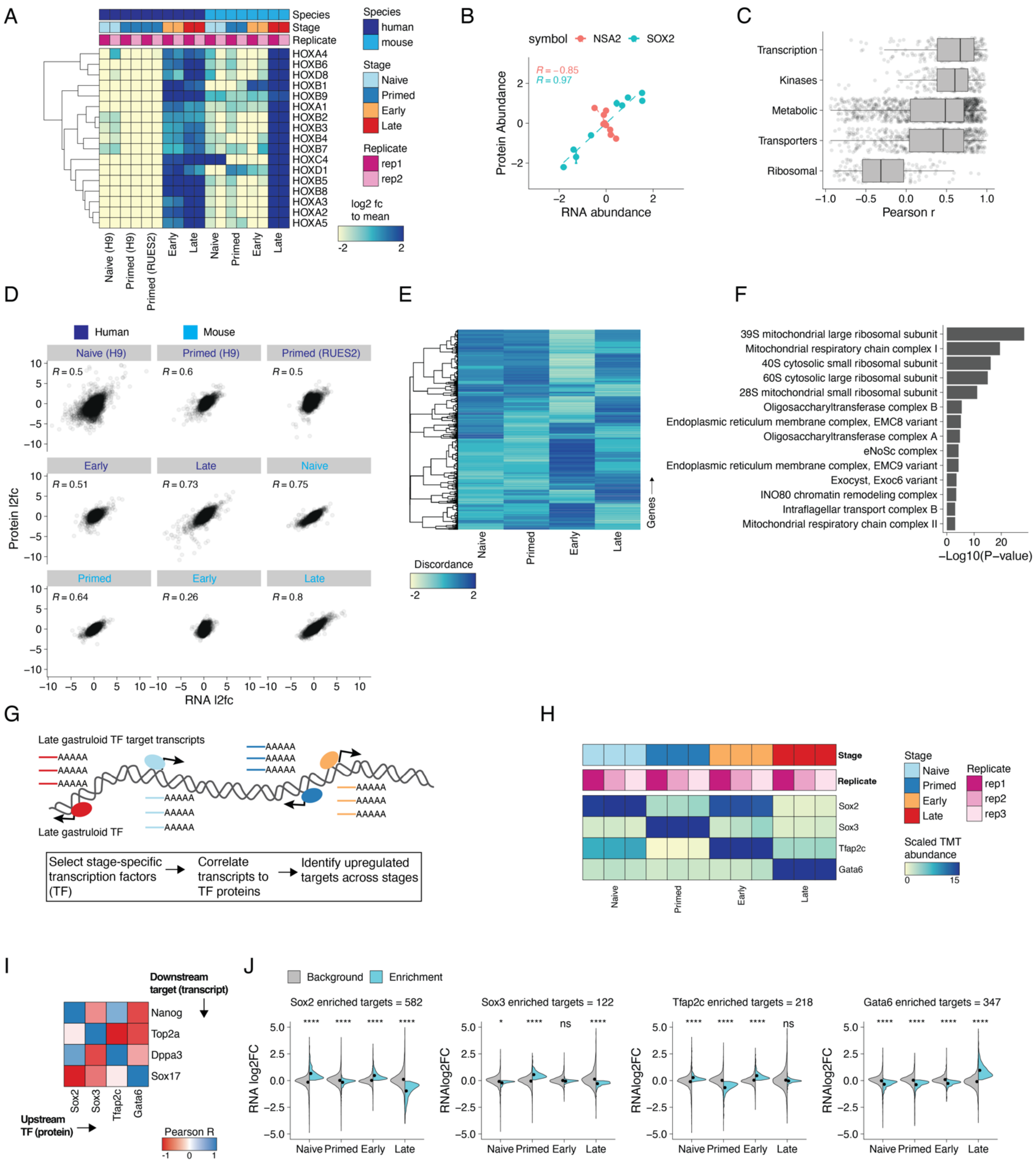

Supplementary Figure 4. Identifying patterns of RNA-protein discordance across gastruloid development. (A) The temporal dynamics of the HOX gene expression cluster. Rows indicate genes, while columns signify samples. Color scale represents the log2 fold change of transcripts normalized to each sample's respective species mean. **(B)** Representative examples of RNA vs. protein abundance correlation for SOX2 (red) and LAMTOR2 (teal). **(C)** Distributions of RNA-protein correlations ($r_{Pearson}$) for gene sets grouped by protein class (curated from Human Protein Atlas). **(D)** Scatterplot of RNA (x-axis) and protein (y-axis) abundance across stages of mouse or human gastruloid development. **(E)** Hierarchical clustering of patterns of RNA-protein discordance ratios across genes during mouse gastruloid development. **(F)** Protein complexes whose RNA abundances differ significantly from their protein abundances when comparing early vs. late

bioRxiv preprint doi: https://doi.org/10.1101/2024.09.05.609098; this version posted September 7, 2024. The copyright holder for this preprint (which was not certified by peer review) is the author/funder, who has granted bioRxiv a license to display the preprint in perpetuity. It is made available under aCC-BY 4.0 International license.

mouse gastruloids. **(G)** Workflow for identifying putative downstream targets of stage-specific transcription factors. **(H)** Stage-specific protein expression of Sox2, Sox3, Tfap2c and Gata6. **(I)** Representative heatmap depicting the $r_{Pearson}$ correlation coefficients of transcription factor protein abundance (columns) to downstream target transcripts (rows) **(J)** RNA abundance distributions (y-axes) of target transcripts to aforementioned transcription factors (top). Colors indicate the enriched (cyan) or background (gray) target transcripts to the corresponding transcription factor. Significance estimated using ANOVA (n.s. denotes not significant; * denotes $p < 0.05$; **** denotes $p < 1.3e-8$).

bioRxiv preprint doi: https://doi.org/10.1101/2024.09.05.609098; this version posted September 7, 2024. The copyright holder for this preprint (which was not certified by peer review) is the author/funder, who has granted bioRxiv a license to display the preprint in perpetuity. It is made available under aCC-BY 4.0 International license.

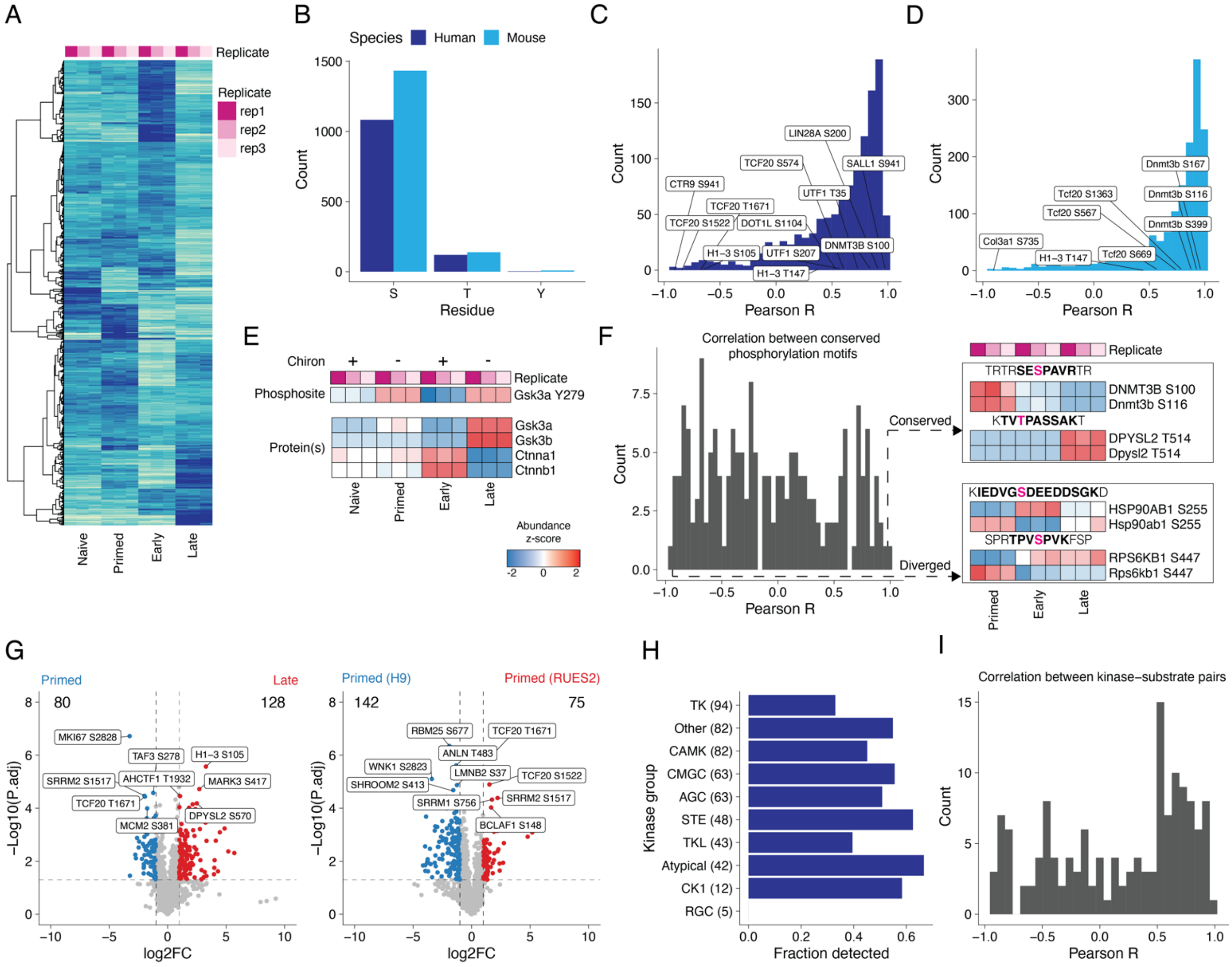

Supplementary Figure 5. Mapping phosphorylation states across gastruloid development. (A) The temporal dynamics of phosphorylated peptides across mouse gastruloid development. **(B)** Number of phosphorylated sites (y-axis) identified per amino acid residue (x-axis). **(C-D)** Distribution of Pearson correlation coefficients ($r_{Pearson}$) from comparing the abundances of phosphosites to their respective (C) human or (D) mouse proteins. **(E)** Effects of temporal Chiron treatment on protein and/or phosphorylation dynamics of Gsk3a, Gsk3b, Ctnna1, and Ctnnb1. **(F)** Distribution of $r_{Pearson}$ computed from comparing temporal abundances of conserved phosphorylation motifs between human and mouse (left). Representative tile plots of conserved and diverged phosphosite profiles across motifs shared between humans and mice (right). Detected peptide (bold) and phosphorylated residue (magenta) are highlighted above each tile plot. **(G)** Volcano plots comparing differentially expressed phosphosites between primed RUES2-GLR vs. late gastruloids (left) or between H9 vs. RUES2-GLR primed ESCs (right). X-axis represents the $\log_2$ fold change between 2 samples and the y-axis represents the negative $\log_{10}$ of the BH-adjusted p-value. **(H)** Proportion of human protein kinases detected by kinase group. Kinase annotations curated from KinMap explorer[111]. **(I)** Histogram of $r_{Pearson}$ between human kinase-substrate pairs detected across human gastruloid development. Pairs curated from PhosphositePlus.

bioRxiv preprint doi: https://doi.org/10.1101/2024.09.05.609098; this version posted September 7, 2024. The copyright holder for this preprint (which was not certified by peer review) is the author/funder, who has granted bioRxiv a license to display the preprint in perpetuity. It is made available under aCC-BY 4.0 International license.

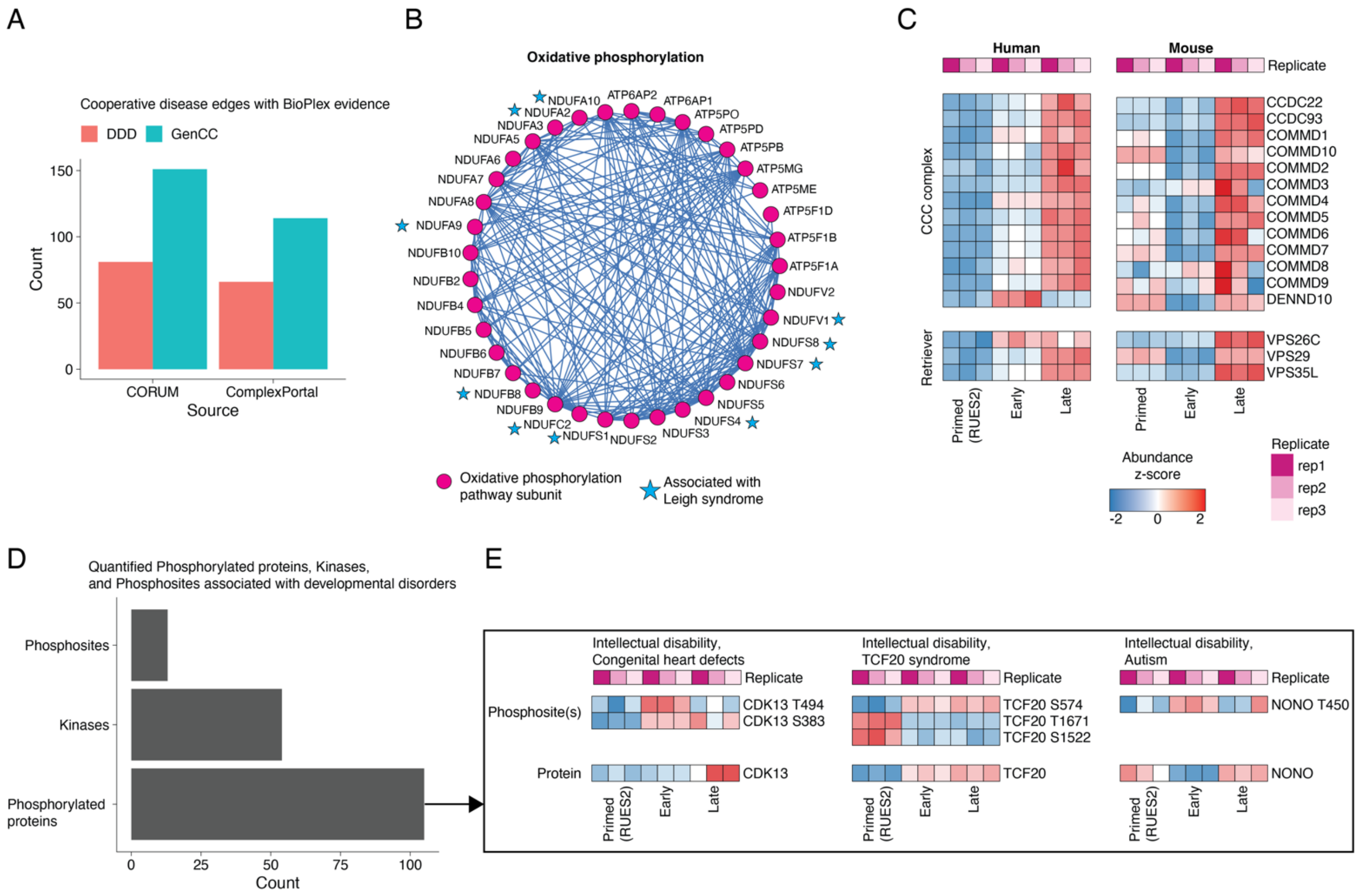

Supplementary Figure 6. (A) Number of cooperative disease proteins with physical evidence in BioPlex or BioGrid to known protein complexes**. (B)** Oxidative phosphorylation co-regulatory network (pathway associations curated from WikiPathways). Proteins associated with Leigh syndrome (blue stars) were enriched in the oxidative phosphorylation co-regulation network (Pathway curated from WikiPathways). **(C)** Temporal protein profiles of the Commander complex across human and mouse gastruloid development. **(D)** Histogram of quantified phosphopeptides, kinases and phosphorylated proteins associated with developmental disorders. **(E)** Temporal phosphorylation states and protein profiles of CDK13, TCF20, and NONO broadly associated with intellectual disability and related developmental disorder.
